# Overlapping roles of spliceosomal components SF3B1 and PHF5A in rice splicing regulation

**DOI:** 10.1038/s42003-021-02051-y

**Published:** 2021-05-05

**Authors:** Haroon Butt, Jeremie Bazin, Sahar Alshareef, Ayman Eid, Moussa Benhamed, Anireddy S. N. Reddy, Martin Crespi, Magdy M. Mahfouz

**Affiliations:** 1 Laboratory for Genome Engineering and Synthetic Biology, King Abdullah, University of Science and Technology (KAUST), Thuwal, Saudi Arabia; 2 CNRS, INRA, Institute of Plant Sciences Paris-Saclay IPS2, Univ Paris Sud, Univ Evry, Univ Paris-Diderot, Sorbonne Paris-Cite, Universite Paris-Saclay, Orsay, France; 3 Department of Biology and Program in Cell and Molecular Biology, Colorado State University, Fort Collins, CO USA

**Keywords:** Plant molecular biology, RNA splicing

## Abstract

The SF3B complex, a multiprotein component of the U2 snRNP of the spliceosome, plays a crucial role in recognizing branch point sequence and facilitates spliceosome assembly and activation. Several chemicals that bind SF3B1 and PHF5A subunits of the SF3B complex inhibit splicing. We recently generated a splicing inhibitor-resistant SF3B1 mutant named *SF3B1* *GEX1A* *RESISTANT 4* (*SGR4*) using CRISPR-mediated directed evolution, whereas splicing inhibitor-resistant mutant of *PHF5A* (Overexpression-PHF5A GEX1A Resistance*, OGR*) was generated by expressing an engineered version PHF5A-Y36C. Global analysis of splicing in wild type and these two mutants revealed the role of SF3B1 and PHF5A in splicing regulation. This analysis uncovered a set of genes whose intron retention is regulated by both proteins. Further analysis of these retained introns revealed that they are shorter, have a higher GC content, and contain shorter and weaker polypyrimidine tracts. Furthermore, splicing inhibition increased seedlings sensitivity to salt stress, consistent with emerging roles of splicing regulation in stress responses. In summary, we uncovered the functions of two members of the plant branch point recognition complex. The novel strategies described here should be broadly applicable in elucidating functions of splicing regulators, especially in studying the functions of redundant paralogs in plants.

## Abstract (web summary)

Butt et al. used CRISPR-mediated directed evolution to generate rice mutants for the spliceosome components SF3B1 and PHF5A. They demonstrate that these mutants have different levels of sensitivity to salt treatments and suggest that the strategies they employed can be used in the future to study functions of redundant paralogs in plants.

## Introduction

RNA splicing is an essential process in eukaryotes whereby the spliceosome excises the intron sequences and ligates the exon sequences together to produce mature mRNAs. The spliceosome is a macromolecular megaDalton machine comprising of small nuclear RNA molecules (snRNAs U1, U2, U4, U5, and U6) and associated proteins. The spliceosome undergoes a complex assembly process, performs two sequential transesterification reactions, and is regulated in response to cellular, developmental, and environmental cues^1,2^. The pre-mRNA contains consensus sequences to identify exon/intron junctions and *cis*-sequences to guide the splicing machinery and other *trans*-factors for successful splicing. Conserved *cis*-splicing consensus sequences include the 5′ and 3′ splice sites (SS), a polypyrimidine tract and the branch point sequence (BP), which are required for recognition and spliceosome assembly via numerous RNA–RNA, RNA–protein, and protein–protein interactions. Moreover, the spliceosome undergoes dynamic conformational rearrangements to mediate the splicing process^3,4^. Splicing must exhibit high fidelity as a single base pair shift in the SS can lead to a coding sequence frameshift that ultimately disrupts translation. Thus, aberrant splicing causes many genetic diseases in humans^5^ and affects a plethora of stress responses in plants^6^. For example, erroneous recognition and selection of the BP can lead to the accumulation of aberrant transcripts in the cell, potentially leading to cancerous phenotypes^7,8^. Alternative splicing (AS), which involves the inclusion or skipping of exonic or intronic sequences and differential selection of 5′ or 3′ SSs, enhances mRNA coding capacity^9,10^ and also regulates the levels of functional mRNAs. AS constitutes a co-/post-transcriptional regulatory layer that is activated by growth, developmental, and environmental cues to ensure appropriate molecular responses^10,11^.

The SF3B complex of the U2 snRNP contains several spliceosome-associated proteins (SAPs), namely SF3B1/SAP155, SF3B2/SAP145, SF3B3/SAP130, SF3B4/SAP49, SF3B5/SAP10, SF3B6/SAP14a, and PHF5A/SF3B7/SAP14b, and this heptameric complex is essential for splicing^8,12^. The SF3B complex is mainly involved in recognition of branch point adenosine (BPA) and promotion of stable interaction of U2 with pre-mRNA^13^. This complex is a target of natural compounds with antitumor activities, including Herboxidiene (GEX1A)^14^, Spliceostatin A (SSA)^15^, and Pladienolide B (PB)^16,17^. These compounds have anticancer activities due to their preferential cytotoxic effects on cancer cells. Structural and biochemical studies have shown that these compounds bind to the SF3B1–PHF5A complex, occupying the pocket of the SF3B complex and blocking SF3B complex binding to the BP^18–20^. As a result, these splicing modulators perturb BP recognition and selection, preventing the stable formation of the U2 snRNP–BP duplex^18,21^. These splicing modulators share common binding sites in SF3B1 and PHF5A proximal to the binding pocket of the BP^18,19,22^. SF3B1 plays a role in the recruitment or stabilization of the U2 snRNP complex at the BP sequence during the formation of the spliceosome A complex^23^. SF3B1 (SF3b155) is the largest subunit of the SF3B complex and contains an unstructured N-terminal domain (NTD) and C-terminal HEAT repeat domain (HD). The NTD and HD play essential roles at different stages of the splicing process^24,25^. The splicing factor PHF5A, characterized by its PHD-domain, is a ubiquitously expressed nuclear protein that is highly conserved among eukaryotes^26^. PHF5A plays an essential role during embryo formation and tissue morphogenesis by modulating pluripotency and cellular differentiation^27^. Dysregulation of PHF5A has different effects on the progression of different cancers^28–30^. PHF5A is a component of the SF3B complex, essential for spliceosome structural stability, and can link the spliceosome to histones^18,29^. Point mutations in key residues of the SF3B complex subunits, including SF3B1-K1071E, SF3B1-R1074H, SF3B1-V1078A/I, and PHF5A-Y36C, confer tolerance to chemical compounds that affect splicing^18–20,22^.

In plants, chemical modulators of splicing such as GEX1A and PB affect the splicing machinery resulting in genome-wide inhibition of splicing^31,32^. Although they have considerable effects on all modes of splicing, these compounds show some level of specificity for stress-related genes. Furthermore, in plants, functions of SF3B1 and PHF5A subunits of the SF3B complex, the known targets of inhibitors of splicing are largely unknown. Therefore, the use of splicing modulators may assist in understanding the functions of SF3B1 and PHF5A, molecular mechanisms underlying stress responses and splicing regulation in plants^31,32^. Recently, we employed the CRISPR-Cas9 system for targeted engineering of the splicing protein SF3B1 in rice (*Oryza sativa*) under selective pressure^33^. In this CRISPR-directed evolution approach, we recovered different *SF3B1* variants capable of conferring tolerance to the splicing inhibitors GEX1A and PB^33^. The mutant lines carrying these *SF3B1* variants were termed SGR (SF3B1 GEX1A Resistant). However, the global impact of these *SF3B1* mutant variants on gene expression and splicing, as well as the molecular responses of these mutant variants to splicing modulators, were not analyzed in our previous work. Similarly, our understanding of the molecular function of PHF5A, a SF3B1 interactor, in splicing is based primarily on studies in mammalian cell lines. Hence, the roles of PHF5A and SF3B1 in splicing regulation remains largely unknown in plants.

In this study, we determined the molecular function and physiological roles of these two proteins of the branch point recognition complex in plants. We report the detailed phenotypic and molecular analyses of the *SF3B1* mutant variant SGR4, insensitive to splicing modulators. Compared with WT plants, SGR4 did not exhibit disturbed pre-mRNA splicing under splicing inhibition by GEX1A. Moreover, we also engineered *OsPHF5A* to become resistant to the splicing inhibitory drug and showed that heterologous expression of PHF5A-Y36C in rice confers tolerance to splicing modulators. Global analysis of splicing in wild-type and these two mutants in the presence and absence of a splicing inhibitor revealed the role of SF3B1 and PHF5A in splicing regulation and its impact on rice stress responses. We discovered that the retained introns associated with the inhibition SF3B1 and PHF5A activity are shorter, have higher GC content, and have shorter and weaker polypyrimidine tracts. The GO terms enriched under splicing inhibition conditions are mainly ‘response to chemical’ and ‘response to stress’. Furthermore, splicing inhibition increased seedlings sensitivity to salt stress. Collectively, our results uncovered the functions of two members of the branch point recognition complex. These novel approaches should be largely useful in revealing functions of splicing regulators and to study the role of redundant homologs in plants under normal and stress conditions.

## Results

### SGR4 displays insensitivity to the splicing-inhibitor GEX1A

**Fig. 1:**
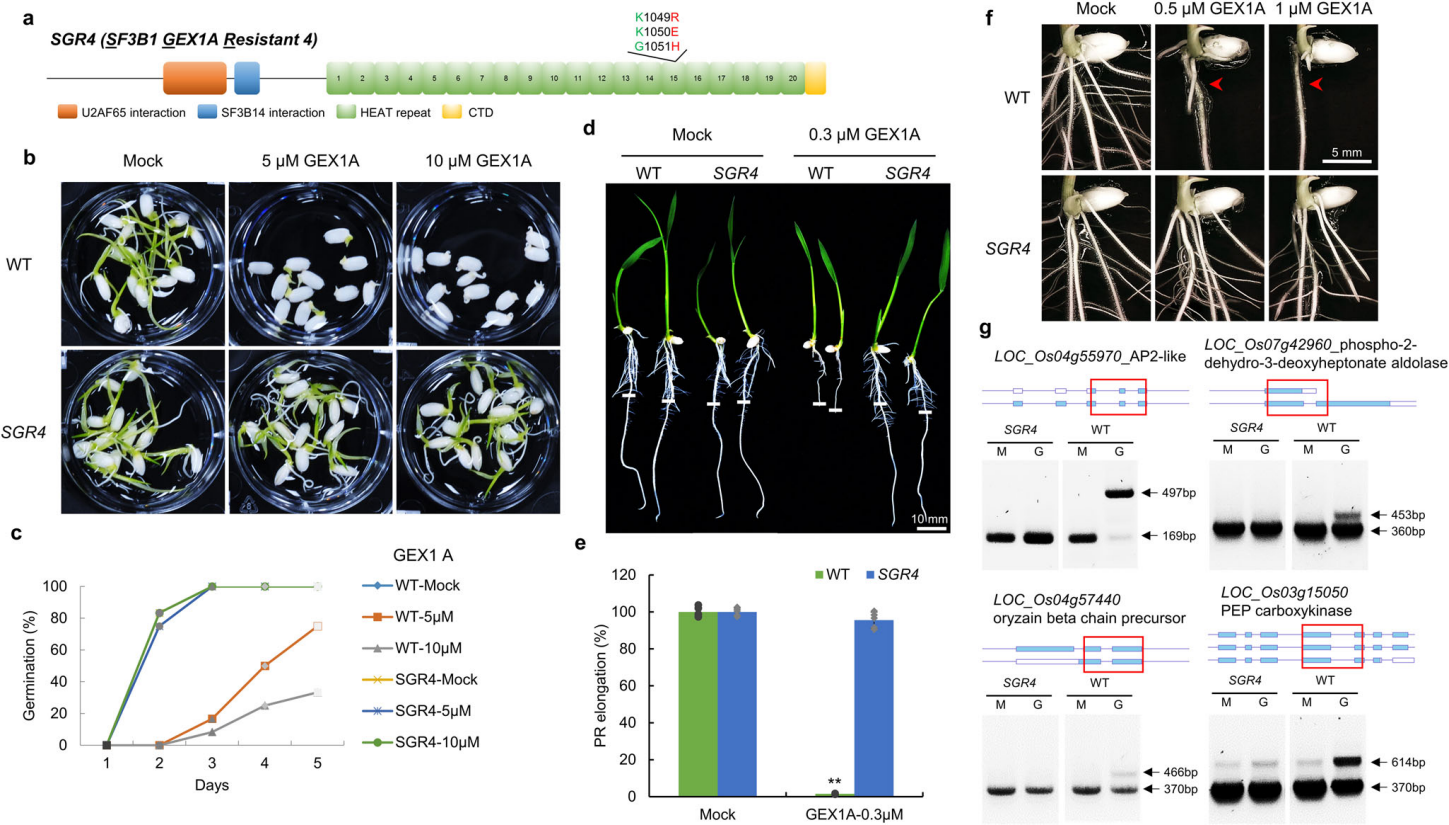
*SGR4* exhibits insensitivity to splicing inhibition under GEX1A treatment. **a** SGR4, an SF3B1 mutant, was evolved through the CRISPR-based directed evolution (CDE) platform, has coding changes of c3144t, a3145c, a3146g, g3147t, a3148g, g3150a, g3151c, g3152a, t3153c and AA changes K1049R, K1050E, G1051H. **b**, **c** Seeds of *SGR4* and WT were germinated for five days in water supplemented with 0, 5, and 10 µM GEX1A. The emergence of plumule was considered as germination. The germination is not only delayed but the shoot and root growth is severely inhibited in WT seeds. *SGR4* germination was not affected by GEX1A treatment. **d**, **e** Seedlings of *SGR4* and WT were germinated for three days on ½ MS media and then transferred to ½ MS media supplemented with 0.3 µM GEX1A for a further three days. Root tips were marked after transfer to new media to observe growth. The root growth of WT seedlings is seized while *SGR4* is unaffected. (*n* = 5). **f** Seedlings were germinated for three days and later transferred to ½ MS media supplemented with 0.5 µM or 1 µM GEX1A. Lateral root growth was observed under a microscope. Lateral roots of WT are severely inhibited whereas the roots of SGR4 are insensitive. **g** cDNA was prepared from 1-week-old rice seedlings following treatment with 0.3 μM GEX1A for 6-h. RT-PCR was performed using primers that flank introns subject to AS in selected genes. Intron inhibition is observed in WT rice plants under GEX1A treatment. Arrowheads indicate splicing variants that changed following GEX1A treatment. The gene structures and retained introns are shown. Red boxes indicate the PCR fragments. M Mock treatment, G GEX1A treatment.

The SF3B1 protein has U2AF65 interaction and SF3B14 interaction domains in the N-terminal region and HEAT repeat domain (HD) and CTD domains in the C-terminal region (Fig. 1a). SGR4 was generated using CRISPR-mediated directed evolution platform and carries K1049R, K1050E, G1051H substitutions (Fig. 1a). We have previously shown that SGR4 is resistant to the GEX1A^33^. To investigate the effect of GEX1A on the growth and development of SGR4, we conducted a detailed phenotypic analysis. We applied different concentrations of GEX1A to WT and the SGR4 and observed the effects on seed germination and seedling growth. Our analysis indicated that the germination of SGR4 is not affected even at 10 µM GEX1A while WT germination is severely inhibited at 5 µM GEX1A (Fig. 1b, c). Consistent with the germination assays, SGR4 has a sustained primary root length in the presence of 0.3 μM GEX1A whereas WT was completely arrested (Fig. 1d, e). Next, we investigated the effect of the GEX1A splicing modulator on lateral root growth in WT and SGR4. We conducted a lateral root assay using 0.5 μM and 1 μM GEX1A treatments. The WT plants exhibited sensitivity to 0.5 μM and 1 μM GEX1A treatments, leading to inhibition of lateral root formation, confirming that splicing regulation is an important component of LR formation and development. However, SGR4 shows increased LR density, manifested as complete insensitivity to the GEX1A treatments. (Fig. 1f). These data indicate that the SGR4 may have different structural features^33^ that affect its binding to the GEX1A splicing modulator, resulting in GEX1A-insensitive phenotypes in seed germination, primary root length, and lateral root growth.

GEX1A and other splicing modulators inhibit splicing leading to considerable levels of intron retention (IR) in plants^31,32^. We hypothesized that the GEX1A-tolerance phenotype was linked to splicing regulation. In our previous study, we have tested the SGR4 exhibited GEX1A-resistant molecular phenotypes on IR and splicing inhibition^33^. To show that this effect is wider and applicable to a large set of genes, we selected another group of rice genes known to undergo AS and used RT-PCR to determine the level of IR under GEX1A treatment. Specifically, RT-PCR primer sets were designed for genes encoding the AP2-like ethylene-responsive transcription factor AINTEGUMENTA (*LOC_Os04g55970*), oryzain beta chain precursor (*LOC_Os04g57440*), phosphoenolpyruvate carboxykinase (*LOC_Os03g15050*), and phospho-2-dehydro-3-deoxyheptonate aldolase, chloroplast precursor (*LOC_Os07g42960*). We treated 1-week-old seedlings of WT and the SGR4 mutant with 0.3 μM GEX1A for 6 h. Subsequently, using cDNA from each line, we performed RT-PCR targeting exons encompassing the intron involved in an AS event. Our RT-PCR data corroborate our phenotypic data showing that AS in SGR4 is insensitive to GEX1A treatment and remains similarly efficient as the AS observed under control conditions suggesting that the splicing-dependent gene expression pattern induced by the drug can be overcome by the SGR4 mutation (Fig. 1g).

These data link the GEX1A-tolerance phenotype to splicing and indicate that the structure of the SF3B1 variants and their binding to the GEX1A splicing modulator determine the resulting splicing efficiency and response to splicing inhibition phenotypes. Therefore, the SGR4 mutant provides a valuable resource to investigate the molecular mechanisms underlying splicing inhibition by splicing modulators and its impact on plant growth phenotypes.

### SGR4 inhibits GEX1A-induced change in global gene expression and splicing

**Fig. 2:**
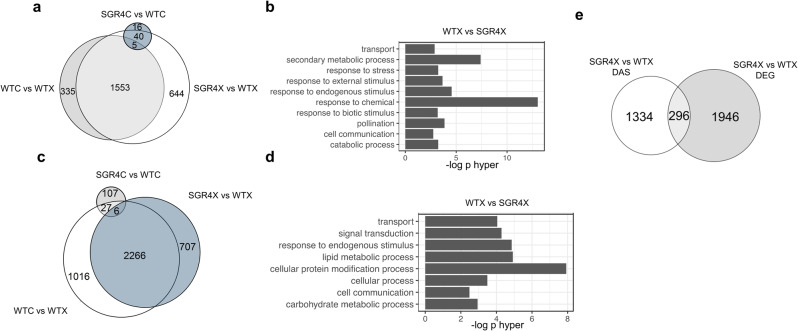
*SGR4* protects against GEX1A-induced gene expression and splicing inhibition. **a** Overlap of differentially expressed genes (DEGs) between WT and *SGR4* under mock or GEX1A treatment. One-week-old rice seedlings were treated with 0.3 μM GEX1A for 6 h. A high number of DEGs were observed after GEX1A treatment in WT while *SGR4* appears insensitive to GEX1A. *SGR4* comparison with WT under mock treatment show very few number of DEGs. **b** GO enrichment analysis of the DEGs regulated by GEX1A. **c** Venn diagram showing the overlap of differentially spliced events (DAS) between WT and *SGR4*. **d** GO enrichment of GEX1A-responsive DAS genes. **e** Overlap of DEG with DAS events for SGR4 and WT under GEX1A treatment.

To determine the effect of the *SF3B1* mutations in SGR4 on genome-wide gene expression and pre-mRNA splicing, we performed RNA-seq analysis of 1-week-old rice seedlings of WT and SGR4 using polyA+ RNA. We identified 68 differentially expressed genes (DEGs) under control conditions (Fig. 2a, Table 1).

**Table 1:** Number of DEG per comparision.

Comparison	Direction	DEG number
SGR4X vs SGR4C	Up	17
	Down	4
WTC vs SGR4C	Up	21
	Down	47
WTX vs SGR4X	Up	1115
	Down	1127
WTX vs WTC	Up	1041
	Down	859
OGR1X vs OGR1C	Up	207
	Down	217
WTC vs OGR1C	Up	306
	Down	438
WTX vs OGR1X	Up	1272
	Down	663
WTX vs WTC	Up	1345
	Down	736

To further dissect the effect of SGR4 on AS, we analyzed differential splicing between these lines using rMATS. Similarly, to differential expression analysis, SGR4 had a minor impact on all types of splicing events in control conditions (Fig. 2c, Table 2). To investigate the genome-wide effects of SGR4 and the resulting molecular responses to splicing-inhibition, 1-week-old seedlings of WT and SGR4 were treated with 0.3 µM GEX1A for 6 h and RNA deep sequencing was performed to do a similar analysis on differential gene expression and AS. Indeed, our previous studies have shown that GEX1A treatment results in significant inhibition of constitutive splicing and AS^31,32^. Splicing inhibition is likely due to the binding of GEX1A to the SF3B1 proteins and the perturbation of the U2 snRNP machinery. We recently showed that GEX1A may not be capable of binding to the SF3B1 variants in the SGR mutants or that this binding may be compromised, which would thus compromise GEX1A-mediated splicing inhibition^33^. We wanted to determine whether the SF3B1 mutant variant maintains its splicing functions or whether they result in global differential splicing due to its potentially compromised binding to the splicing modulator. As expected, when treated with 0.3 μM GEX1A, WT seedlings exhibit major changes in DEG number (Table 1) and in the retention of a large number (2832) of introns but also on all types of AS events (3′ SS, 5′ SS, and ES) (Table 2). Nevertheless, in line with the above-described phenotypic data, our analysis showed that gene expression was almost unaffected by GEX1A treatment in SGR4. After GEX1A treatment, we detected only 21 DEG (Table 1) and 320 AS events (Table 2) in SGR4, further corroborating our data that SGR4 is largely insensitive to GEX1A. To identify genes whose GEX1A response is affected at DEG and splicing level in WT in comparison to SGR4, we compared at genome-wide level SGR4 and WT under GEX1A treatment. We identified a large number of DEG and DAS events, which show strong overlap with GEX1A-induced DEG and DAS between WT and SGR4 respectively (Fig. 2a, c). Consistent with our previous work showing that GEX1A preferentially affected splicing of stress-related genes^31,32^, GO analysis of GEX1A-regulated DEG also indicated enrichment in transport, secondary metabolic process, response to stress, response to endogenous stimulus, response to chemical, response to biotic stimulus, pollination, and catabolic process (Fig. 2b). At the splicing level, DAS genes were enriched in multiple cellular pathways like cellular protein modification process, response to endogenous stimulus, lipid metabolic process, and signal transduction (Fig. 2d). Although the global GO is in the same pathways, we have observed very little correlation between DEG and DAS events (Fig. 2e), which indicate most of the DEG are not alternatively spliced and vice versa.

**Table 2:** Number and type of AS events per comparison.

Comparison	Type of event	Number of AS events
SGR4C vs SGR4X	A3S	43
	A5S	62
	IR	175
	ES	40
WTC vs SGR4C	A3S	28
	A5S	12
	IR	96
	ES	15
WTX vs SGR4X	A3S	141
	A5S	199
	IR	2603
	ES	143
WTX vs WTC	A3S	181
	A5S	228
	IR	2855
	ES	173
OGRX vs OGRC	A3S	54
	A5S	69
	IR	469
	ES	45
WTC vs OGRC	A3S	152
	A5S	94
	IR	1452
	ES	82
WTX vs OGRX	A3S	170
	A5S	159
	IR	2828
	ES	112
WTX vs WTC	A3S	272
	A5S	300
	IR	3841
	ES	227

Overall, our data confirm that global GEX1A-induced DEG and splicing inhibition is apparent in WT whereas negligible effects on DEGs and DAS are observed in SGR4 following GEX1A treatment, which supports its highly GEX1A-insensitive phenotype.

### Expression of engineered PHF5A-Y36C, another member of the branch point recognition complex, confers tolerance to GEX1A

**Fig. 3:**
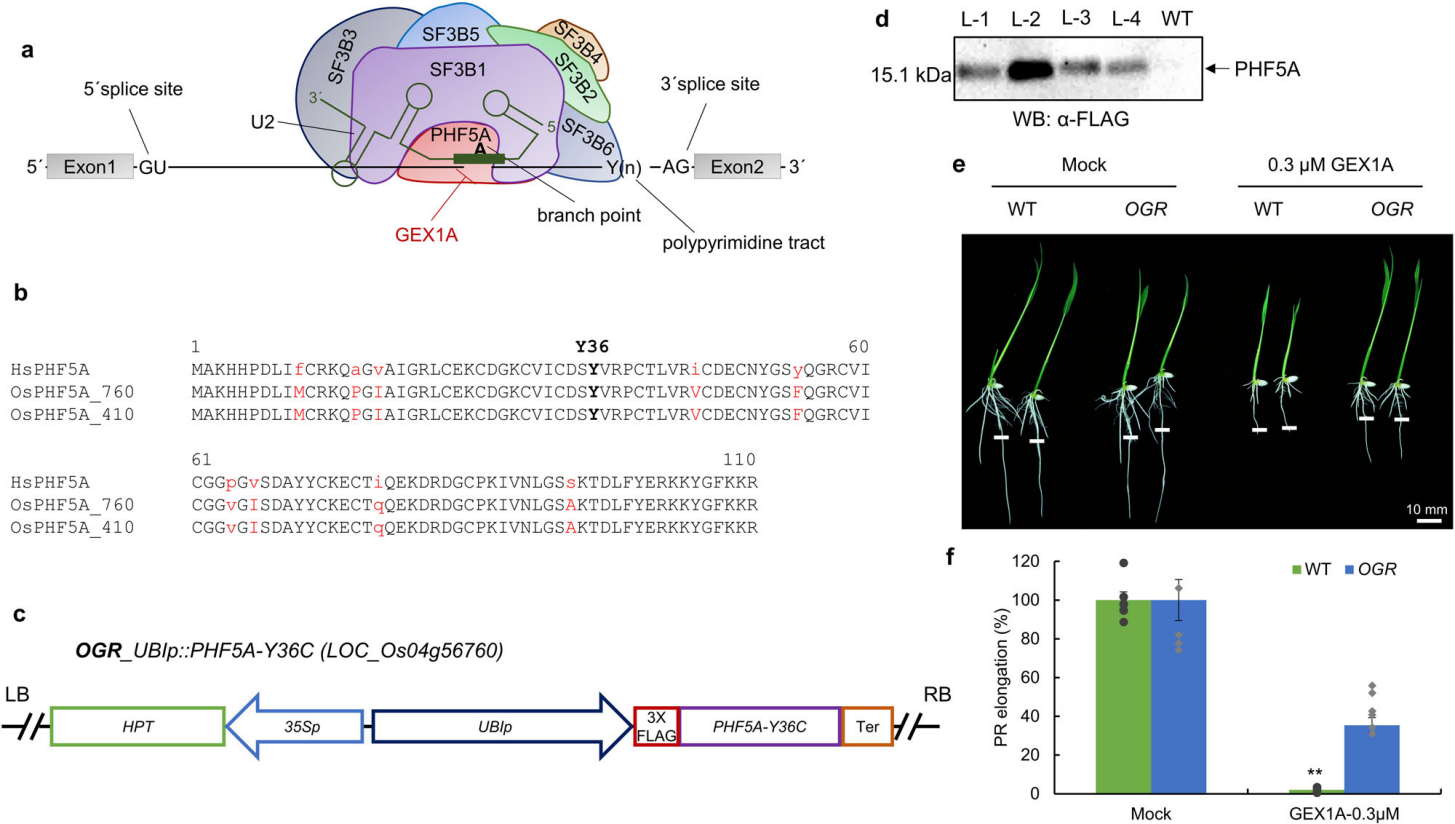
Generation of PHF5A-Y36C heterologous expression in rice. **a** The structure of SF3B complex binding to U2 and the pre-mRNA intron. The branch point is in contact with SF3B1 and PHF5A. The branch point recognition sequence (BPRS) of U2 snRNA binds to branch point and flanking nucleotides. **b** GEX1A interrupts these interactions and inhibit this early spliceosome assembly. The human PHF5A protein sequence is aligned with sequences of rice PHF5A homologs. PHF5A-Y36C (highlighted in bold) confers tolerance to splicing modulators (Teng et al., 2017) and is highly conserved among eukaryotes. **c** The coding sequence of rice *OsPHF5A* (*LOC_Os4g56760*) was mutated (Y36C; TAT to TGT) and cloned into a construct under the control of the Ubiquitin promoter with a 3XFLAG-tag. **d** Confirmation of PHF5A-Y36C heterologous expression in transgenic lines. Total protein was extracted from independent lines of *O. sativa cv japonica* transformed with UBIp::PHF5A-Y36C. Anti-FLAG antibody was used to detect FLAG-tagged PHF5A-Y36C. The arrow indicates the presence of PHF5A. WT was used as a negative control. **e**, **f** Heterologous expression of PHF5A-Y36C confers tolerance to GEX1A. Rice seeds were germinated on 1⁄2 MS media for 3 days, then transferred onto 1⁄2 MS media supplemented with 0.3 μM GEX1A for 3 days. Root tips were marked to observe post-transfer growth. The transgenic lines were termed OGR (Overexpression-PHF5A GEX1A Resistance).

The SF3B complex consists of seven proteins with distinct domain structures and molecular sizes (Fig. 3a)^34^, and none of these proteins is characterized in plants yet. The SF3B1 together with PHF5A recognize and bind the BP during splicing reaction. The SF3B1 HEAT repeats grab the U2/branch point sequence (BPS) duplex and BP Adenosine is bulged out and covered in a pocket between HEAT repeats and PHF5A^35^. This structure permits the SF3b complex to protect the BP from premature nucleophilic attack of its 2´-OH and adopt a proper confirmation before the first transesterification reaction^13^. Splicing inhibitors like GEX1A disrupt the interactions of the SF3B1 and PHF5A with BP and impairs the pre-mRNA splicing^36^. Recently, in an effort to identify clones tolerant to splicing modulators such as E707, whole-exome sequencing data identified a *PHF5A* mutant, specifically PHF5A-Y36C, as a molecular target in mammalian cells conferring tolerance to the E707 splicing modulator^18,22^.

To identify the homolog of human PHF5A in rice, we interrogated the rice genome database and identified two homologous *OsPHF5A* genes (*LOC_Os04g56760* and *LOC_Os05g30410*), which show little variation in DNA sequence and encode identical proteins (Fig. 3b). We named these loci *OsPHF5A1* (*LOC_Os04g56760*) and *OsPHF5A2* (*LOC_Os05g30410*). A protein alignment showed that the majority of protein regions share high sequence similarity between human and rice PHF5A homologs (Fig. 3b). In particular, the Y36 residue is conserved among these protein sequences. We used the *OsPHF5A1* (*LOC_Os04g56760*) DNA sequence and introduced a single nucleotide substitution mutation (TAT to TGT, Y36C) to create PHF5A-Y36C for expression in rice (Fig. 3c). The synthetic PHF5A-Y36C cassette was FLAG-tagged and expressed under the control of the *OsUBIQUITIN* promoter. In the T_0_ plants, PHF5A-Y36C expression was analyzed using FLAG-tag, and confirmed lines were used for further experiments (Fig. 3d).

In the progeny, we examined the effects of the GEX1A splicing modulator on the growth and development of *PHF5A-Y36C-OX* plants. Our phenotypic analysis indicated that these *PHF5A-Y36C-OX* plants were partially tolerant to GEX1A- and PB-treatment (Fig. 3e, Supplementary Fig. 1), which is different from SGR4. We termed this line OGR (Overexpression-PHF5A-Y36C GEX1A Resistance). The overexpression of PHF5A-Y36C had no apparent effect on plant growth and development and OGR plants were phenotypically indistinguishable from WT plants.

To further dissect the role of OsPHF5A in rice growth and splicing, we employed CRISPR/Cas9-targeted mutagenesis to engineer functional knockout of the *OsPHF5A* genes. As we did not find a PAM site to cut exactly at Y36, we designed two sgRNAs closest to the available PAM site to target each of the *OsPHF5A* loci (Supplementary Fig. 2A, B). In the T_0_ generation, we recovered several single knockout mutants. For *OsPHF5A1* (*LOC_Os04g56760*), targeting via sgRNA-1 produced a homozygous monoallelic knockout mutant with two nucleotide deletions that caused a stop codon after K25 (Supplementary Fig. 2A). Similarly, targeting via sgRNA-2 also produced a homozygous monoallelic knockout mutant with one nucleotide insertion that produced a frameshift after C40 and a stop codon (Supplementary Fig. 2A). To target *OsPHF5A2* (*LOC_Os05g30410*), we also used two sgRNAs. Targeting via sgRNA-1 produced a homozygous monoallelic knockout mutant with one nucleotide insertion that caused a frameshift after Y36 and a stop codon (Supplementary Fig. 2B). Using sgRNA-2, we produced a homozygous biallelic knockout mutant. One allele has a single nucleotide deletion and caused a frameshift after Y36 with 38 additional residues. The second allele has one nucleotide insertion and caused a frameshift after V37 and a stop codon (Supplementary Fig. 2B).

We tested the effects of GEX1A on the growth and development of *OsPHF5A* homozygous mutant lines. We treated 3-day-old seedlings with 0.3 µM GEX1A and observed subsequent primary root growth. Our data show that these knockout mutants are partially tolerant to GEX1A treatment (Supplementary Fig. 2C). We termed these *OsPHF5A* mutants PGR (PHF5A GEX1A Resistance). The degree of GEX1A tolerance was similar in all PGR mutants but slightly weaker than that in OGR seedlings (Supplementary Fig. 2C).

Overall, these results indicate that the expression of mutant variant PHF5A-Y36C at a single locus is sufficient to perturb GEX1A binding. Similarly, the two rice *PHF5A* genes may have additive effects and knocking out either of them perturbed the binding with splicing inhibitors. However, it seems that OsPHF5A is essential for plant survival as we did not recover a functional double knockout mutant.

### Expression of PHF5A-Y36C represses GEX1A-mediated splicing inhibition

**Fig. 4:**
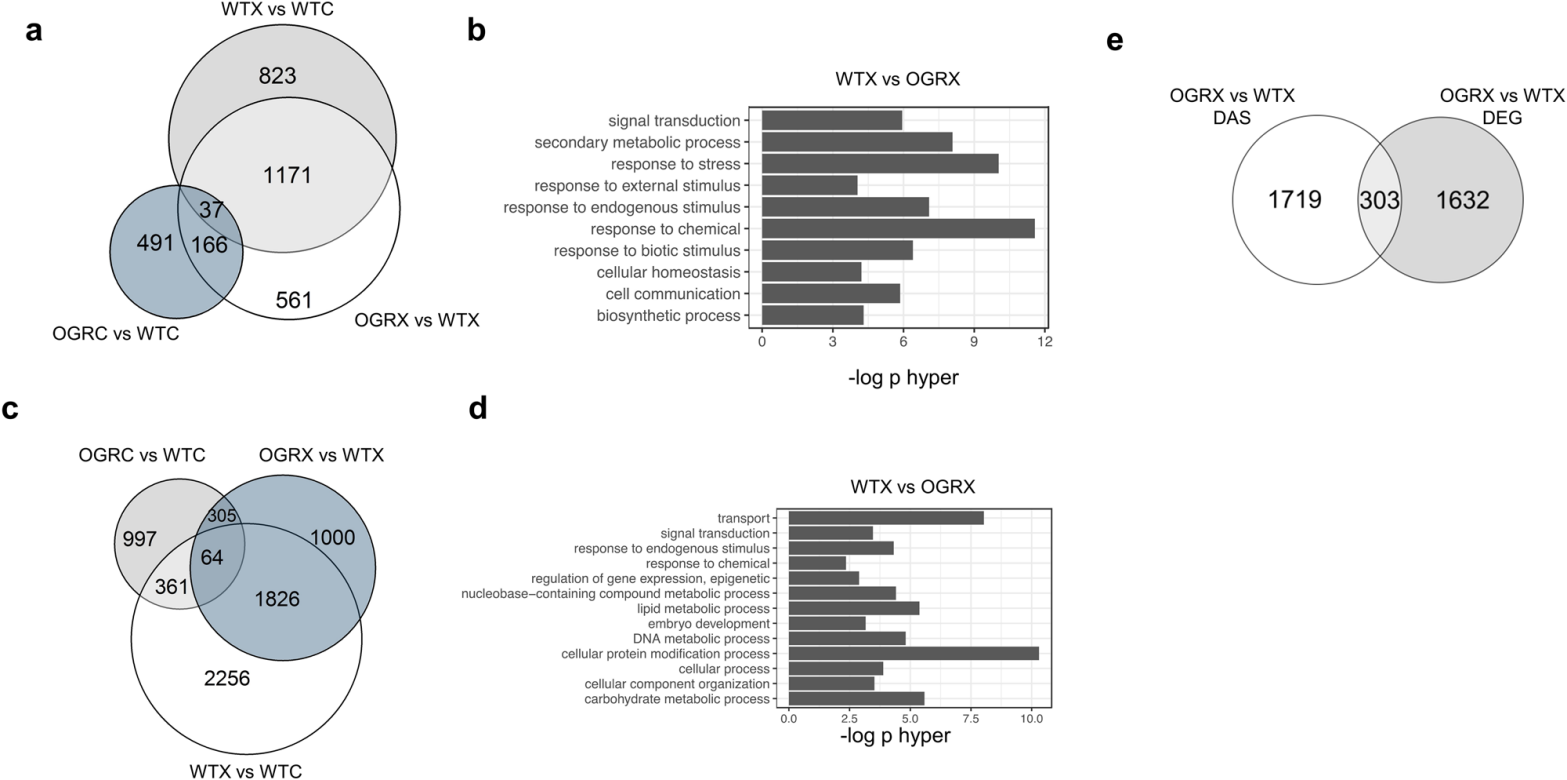
OGR impedes the effects of GEX1A on global gene expression and splicing patterns. **a** Overlap of differentially expressed genes (DEGs) between WT and *OGR* under mock or GEX1A treatment. The number of DEGs much higher in WT compared to *OGR*. Heterologous expression of PHF5A-Y36C affects the global gene expression under mock conditions. **b** GO enrichment analysis of the DEGs regulated by GEX1A. **c** Overlap of differentially spliced events (DAS) between WT and *OGR*. **d** GO enrichment of GEX1A-sensitive DAS genes. **e** Overlap of DEG with DAS events for OGR and WT under GEX1A treatment.

To determine the role of PHF5A in genome-wide pre-mRNA splicing under splicing inhibition, we treated 7-day-old seedlings of WT and OGR with 0.3 µM GEX1A and performed RNA-seq analysis using polyA+ RNA. First, we analyzed gene expression in OGR as compared to WT in control condition. We identified 744 DEGs in OGR (Table 1) and 1780 AS events in OGR as compared to WT in control condition, among which were 152 3′ SS, 94 5′ SS, 82 ES, and 1452 IR genes (Table 2). The comparison of WT and OGR under GEX1A treatment show a high number of DEG (1935) and most of these overlap with the ones identified as GEX1A responsive in WT (Fig. 4a). But the treatment with GEX1A of OGR led to only 424 DEG as compared to mock conditions showing that OGR is less sensitive to GEX1A, in agreement with the phenotyping data (Table 1).

AS analysis showed that OGR exhibited a much lower number of differential splicing events compared to WT following GEX1A treatment, confirming that the Y36C mutation provides tolerance to splicing inhibition by GEX1A (Fig. 4c). The numbers of DAS events were 4640 in WT and only 637 in OGR (Table 2). A large number of DAS events were identified when comparing WT and OGR after GEX1A splicing inhibition. Most of these DAS events overlap with GEX1A-responsive events in WT (Fig. 4c). IR events were the most abundant type of AS event affected by OGR under GEX1A but all types of AS events were also represented. As previously shown for SGR4, the GO analysis shows that most of the DEG identified during comparison of WT and OGR under GEX1A treatment are enriched in response to chemical, response to stress, secondary metabolic process, response to endogenous stimulus and response to biotic stress (Fig. 4b). Whereas, DAS were enriched in genes involved in various functional categories such as cellular protein modification process, transport, carbohydrate and lipid metabolic processes and response to an endogenous stimulus (Fig. 4d). We did not observe a very high correlation between DEG and DAS events and most of these DEG and DAS are unique compared to those under GEX1A treatment conditions (Fig. 4e).

Taken together our data show that splicing-inhibition treatment affects genome-wide gene expression in WT. However, global GEX1A-induced splicing inhibition is clearly evident in WT whereas minimal effects on DEGs and DAS are observed in OGR following GEX1A treatment, which supports its highly GEX1A-tolerant phenotype.

### SF3B1 and PHF5A-regulated genes and AS events showed significant overlap

**Fig. 5:**
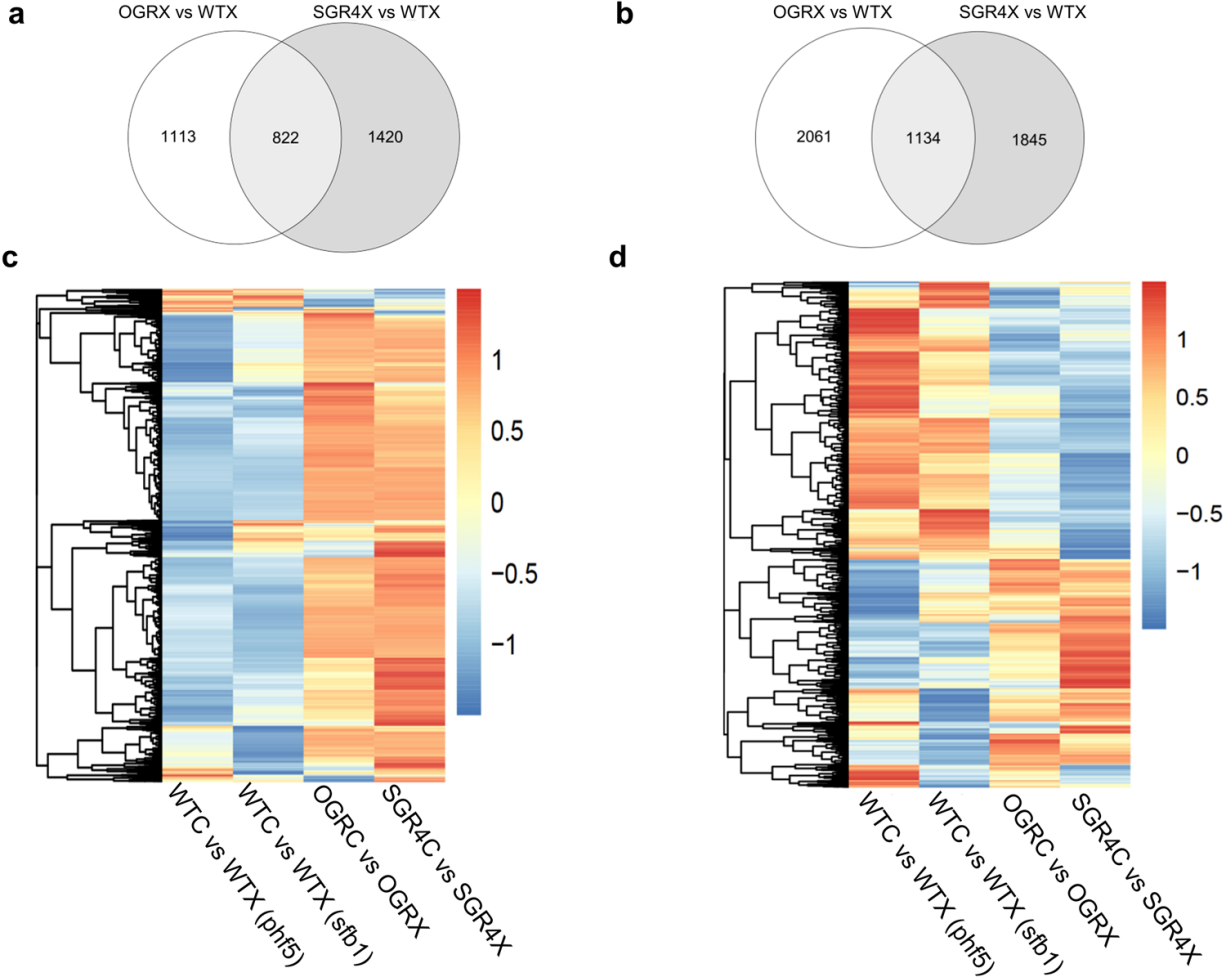
SF3B1- and PHF5A-regulated genes and AS events displayed significant overlap. Overlap of DEGs **a** and DAS events **b** of GEX1A-responsive gene between SGR4 and OGR. Heatmap and hierarchical clustering of log2FC of DEG. Heatmap and hierarchical clustering of log2FC of DEG **c** and delta Ψ (change in splicing) of each intron-retention event **d** significantly affected by GEX1A in WT_sfb1, WT_phf5, SGR4, and PHF5 overexpressing line (OGR). WT of the SF3B1 (WT_sfb1) and PHF5A (WT_phf5).

SF3B1 and PHF5A are partner proteins and function in the SF3B complex as part of the U2 snRNP. The structural and functional affinity emphasizes a common downstream effect on differentially expressed and differentially spliced genes. To identify the similar and differential effect of these partner proteins, we compared the data sets of SGR4, carrying an SF3B1 mutant variant insensitive to GEX1A, with OGR, a PHF5A-Y36C overexpression line highly tolerant to GEX1A. We compared the DEGs and DAS genes significantly affected in GEX1A-treated SGR4 or OGR with those of their respective WT (Fig. 5). This identified the genes that do not respond to GEX1A inhibition in these mutants but that are targeted by GEX1A in WT plants. The data show that OGR and SGR4 are impaired in the GEX1A response involving 822 DEGs and 1134 DAS events (Fig. 5a, b). Heatmap and hierarchical clustering of DEG and splicing of GEX1A-sensitive genes in WT further revealed that SGR4 and OGR similarly affect GEX1A-dependent splicing and DEG in a way that is opposite to what is observed in WT (Fig. 5c, d). However, there is a large number of DAS events that do not overlap (Fig. 5b) which might point toward independent roles during splicing of specific mRNA targets.

**Fig. 6:**
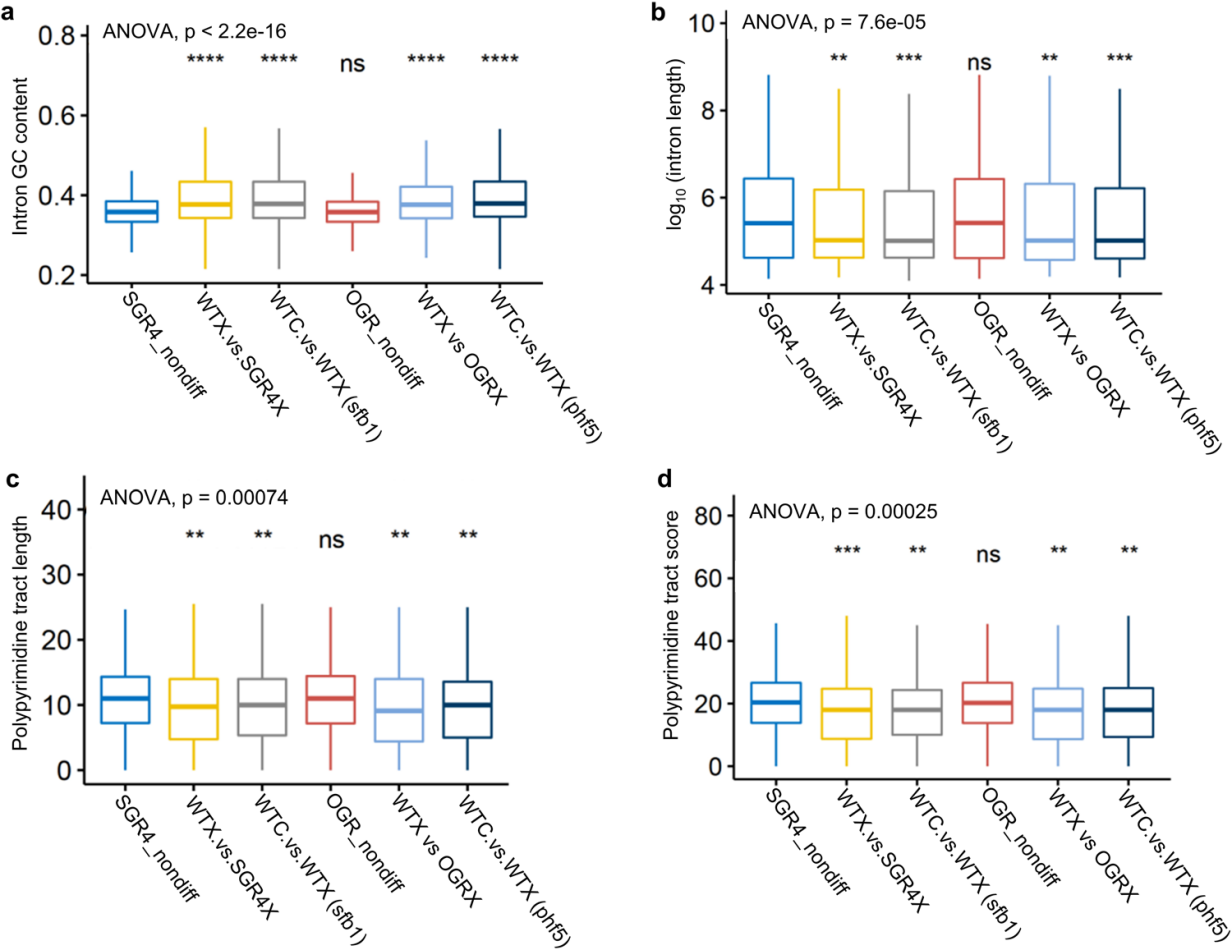
SGR4 and OGR show specificity for splicing-related features. **a** Intron GC content, **b** Intron length, **c** Polypyrimidine tract length, and **d** Polypyrimidine tract score (as defined by SVM-BP, http://regulatorygenomics.upf.edu/Software/SVM_BP/) of introns not affected by GEX1A in SGR4 (SGR4 nondiff) or OGR (PHF5A-Y36C heterologous expressor, OGR_nondiff), introns differentially retained in GEX1A-treated SGR4 or OGR as compared to their corresponding WT (WTX.vs.SGR4X and WTX vs OGRX, respectively) in the same condition, and introns affected by GEX1A in the WT of the SGR4 or OGR experiment (WTC.vs.WTX (sfb1) and WTC.vs.WTX (phf5), respectively). The polypyrimidine tract score indicates C/T content of the polypyrimidine tract between Branch Point and 3′ splice site.

IR results from a pervasive splicing event induced by splicing inhibitors in plants. The AS events are known to be linked with specific splicing elements and nucleotide content, as well as with specific chromatin marks. In particular, intron GC content and the polypyrimidine tract downstream from the BP are important for splicing and may have evolved as recognition signals for the splicing machinery^37,38^. Therefore, we asked whether these sequence elements might also affect splice site recognition in SGR4 or OGR under splicing-inhibition conditions. We compared the sequence elements of retained introns (induced by drug treatment) in WT and SGR4/OGR with those of nonaffected introns in SGR4/OGR. The retained introns have significantly enriched GC content (Fig. 6a). Similarly, the retained introns are shorter in length and displayed significantly weaker Py-tract length and score (Fig. 6b–d). Collectively, these data suggest an important role of these intronic features during GEX1A-mediated splicing inhibition.

### SGR4 and OGR show differing levels of sensitivity to combined salt stress and splicing inhibition

Several reports have linked splicing mutations to sensitivity to abiotic stress factors including salt and drought stress^39–45^. To address the role of SF3B1- and PHF5A-dependent splicing events in relation to abiotic stress, we tested whether SGR4 and OGR had tolerant or sensitive responses to other abiotic stresses, as shown previously in Arabidopsis^31,32^. As mentioned before, GO analysis of GEX1A-induced DAS genes and DEGs revealed that GEX1A targets many abiotic stress-related genes. More specifically, 822 DEG in both SGR4 and OGR lines (Fig. 5a) were significantly enriched for transcription factors GO category including key regulatory players of the salt-stress response in rice such as DREB2A, NAC5, and ERF922^45–48^. The 1134 common AS events (Fig. 5b) were also enriched for important abiotic stress responses such as members of the trehalose biosynthesis pathways which has been linked to salt-stress tolerance in rice^49^.

**Fig. 7:**
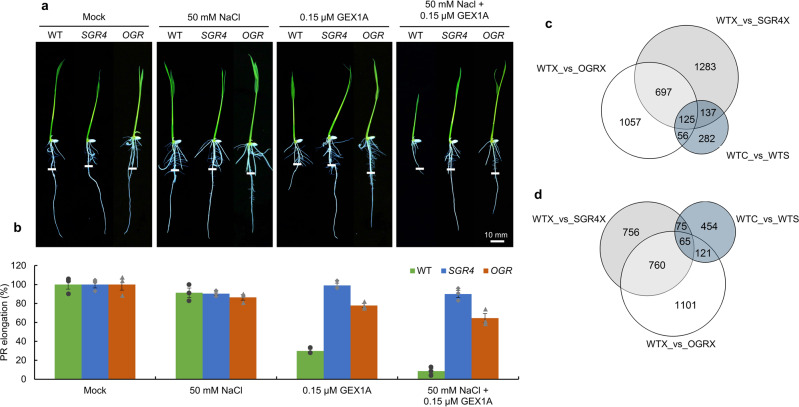
Splicing inhibition sensitizes the WT seedlings against salt stress. **a**, **b** Seedlings of WT, *SGR4*, and *OGR* were germinated for 3 days on ½ MS media and then transferred to ½ MS media supplemented with different concentrations of NaCl and/ or GEX1A for a further 3 days. Root tips were marked to observe post-transfer growth. On 50 mM NaCl or 0.15 µM GEX1A alone, seedling root growth was slightly affected, but severe root growth inhibition was observed in WT seedlings on 50 mM NaCl and 0.15 µM GEX1A combined (*n* = 3). Overlap of DEG **c** and DAS **d** in response to salt treatment (7-day-old WT seedlings treated with 200 mM NaCl for 6-h) in WT (WTC_vs_WTS) with GEX1A-resistant DEG and DAS genes in *SGR4* (WTX_vs_SGR4X) and *OGR* (WTX_vs_OGRX).

Together, this suggested that, the splicing-inhibition treatment mimics an abiotic stress molecular response. We speculated that coupling splicing inhibition with salt stress would induce synergistic effects in stress responses. To test this hypothesis, we subjected 3-day-old seedlings to a 3-day combined treatment of 50 mM NaCl and 0.15 µM GEX1A. Treatment with 50 mM NaCl or 0.15 µM GEX1A alone slightly inhibits root growth in WT. We also tested the primary root growth of WT, OGR, and SGR4 at 100 mM and 200 mM NaCl and did not find any significant differences among these genotypes (Supplementary Fig. 3). However, following combined splicing inhibition at 0.15 µM GEX1A and salt-stress induction at 50 mM NaCl, the primary root growth of WT seedlings was significantly inhibited (Fig. 7a, b). The SGR4 and OGR showed variable levels of tolerance to combined splicing and salt-stress treatment, with OGR exhibiting slight root growth inhibition compared to SGR4, supporting a salt-stress dependent phenotype depending on splicing levels (Fig. 7a, b).

To further validate these responses, we treated the 7-day-old WT seedlings with 200 mM NaCl for 6 h and conducted genome-wide RNA sequencing to identify salt-responsive DEGs and DAS events. We compared the GEX1A-insensitive DEGs and DAS events in SGR4 and OGR to DEG and DAS events in response to salt (Fig. 7c, d). Our analysis showed that 318 out of 600 (53%) of salt-responsive DEGs and 261 out of 715 (36.5%) of salt-responsive AS events overlapped with GEX1A-responsive genes in SGR4 and OGR (Fig. 7c, d).

**Fig. 8:**
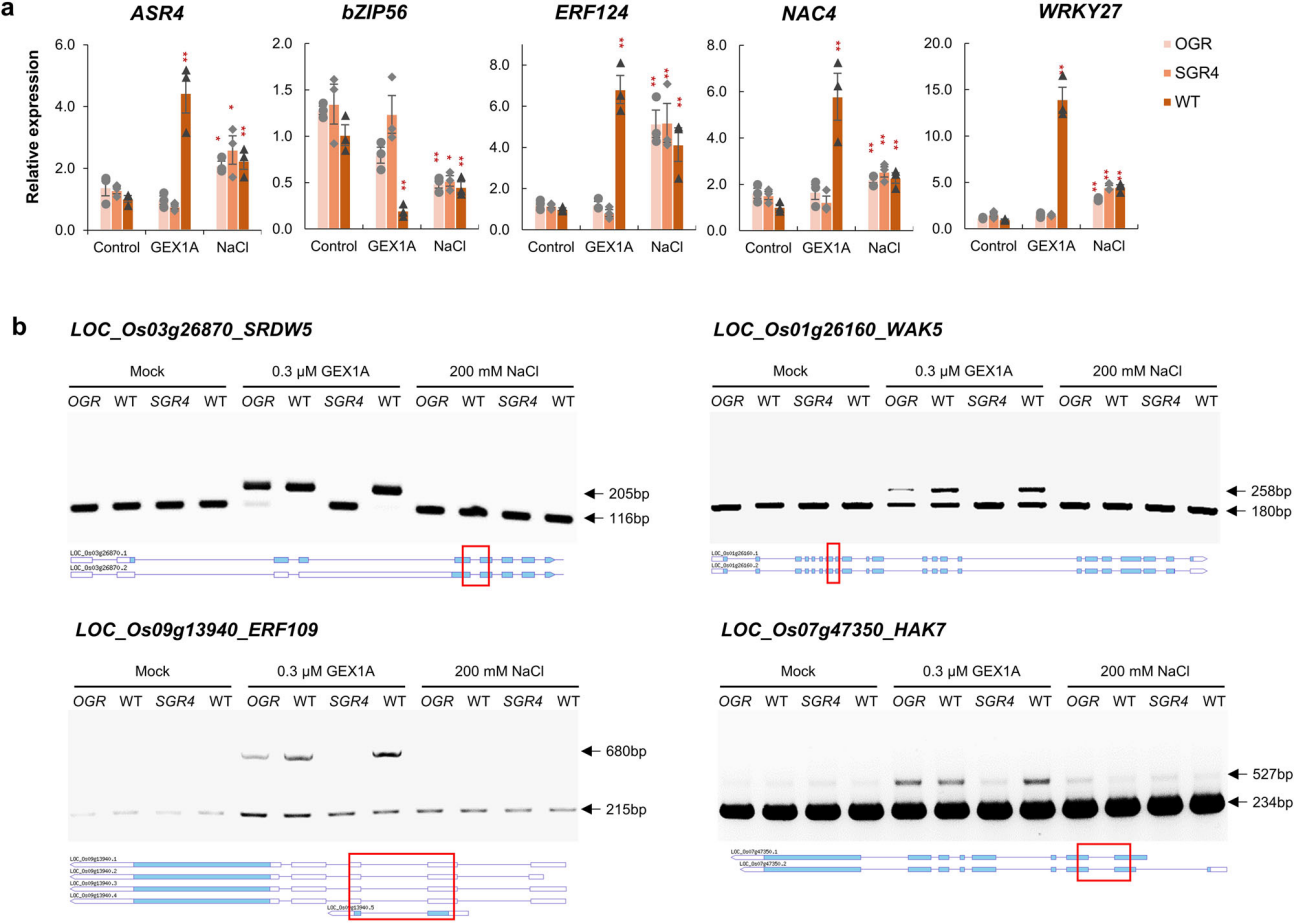
Expression and splicing pattern analysis of stress-responsive genes after GEX1A and salt treatment in OGR and SGR4. One-week-old rice seedlings of OGR, SGR4, and WT are treated with mock, 0.3 μM GEX1A, and 200 mM NaCl for 6-h. Total RNA extracted from the whole seedling was used for mRNA expression and splicing pattern analysis. **a** Expression of stress-responsive genes in OGR, SGR4, and WT. The GEX1A triggers the expression patterns similar to salt-stress treatment for all genes in WT but not in SGR4 and OGR. Bars represent the mean ± SEM of three replicates. *OsActin* was used as an internal control. (Student’s *t*-test; *P < 0.05, **P < 0.01). **b** Semiquantitative RT-PCR analysis of alternative splicing patterns of stress-responsive genes in OGR, SGR4, and WT. No intron retention is observed in SGR4 under GEX1A treatment. Arrowheads indicate splicing variants that changed following GEX1A treatment. The gene structures and retained introns are shown. Red boxes indicate the PCR fragments.

We have previously shown that the splicing inhibition via PB and GEX1A triggers the expression of stress-responsive reporters *RD29A::LUC* and *MAPKKK18::uidA* and activates the ABA signaling pathway in *Arabidopsis*^31,32^. To better understand how the activation of GEX1A regulates stress signaling in rice, we investigated the transcriptional regulation of stress-responsive genes under GEX1A treatment. One-week-old seedlings of OGR, SGR4, and WT were treated with 0.3 µM GEX1A, 200 mM NaCl, and Mock for 6 h. The expression levels of rice stress-responsive transcription factors (*OsASR4*, *OsbZIP*, *OsERF*, *OsNAC*, *OsWRKY*) and several other genes were tested (Fig. 8a, Supplementary Fig. 4). These analyses showed that the GEX1A activates the expression of the stress-responsive genes similar to salt-stress treatment. The transcript levels of *OsASR4*, *OsERF124*, *OsNAC4*, and *OsWRKY27* were significantly elevated, while the transcript level of *OsbZIP* was significantly reduced in WT after GEX1A treatment similar to a salt stress (Fig. 8a). The expression levels of these transcripts were not affected in the OGR and SGR4 after GEX1A treatment (Fig. 8a, Supplementary Fig. 4). Interestingly, the expression of all stress-responsive genes was altered in OGR, and SGR4 like WT under salt-stress treatment. These results show that GEX1A triggers the expression of stress response in rice, a response lost in OGR and SGR4 mutants after GEX1A treatment only because of the inability to bind the GEX1A. This is further verified by the splicing patterns analysis of stress-responsive genes in OGR and SGR4 after GEX1A treatment (Fig. 8b, Supplementary Fig. 5). We selected a set of genes whose splicing is inhibited by the GEX1A in WT rice plants. In our analysis, we did not observe splicing inhibition in SGR4 for any target gene corroborating the GEX1A-insensitive phenotype of SGR4 (Fig. 8b, Supplementary Fig. 5). In OGR the intron retention is reduced for *OsSRDW*, *OsWAK5* and *OsERF109* as compared to wild-type. We did not observe the splicing inhibition after salt treatment for these sets of genes. Together, these data indicate that GEX1A inhibits the splicing of stress-responsive genes and the GEX1A splicing-inhibition overlay salt-stress responses, highlighting a link between splicing and abiotic stress responses in plants that is dependent on the SF3B-PHF5A complex. The generation of gain of function mutants allowed us to detect this overlay stress response linked to splicing regulation in plants.

## Discussion

### SGR4 showed insensitivity to splicing-inhibitor GEX1A

Structural studies have revealed that the SF3B complex plays a role during BP recognition^12,18,23,38,46^. The interaction of SF3B1 HEAT domains and BP adenosine activates the first catalytic step of the splicing reaction^47,48^. These HEAT domain repeats are the main target of small molecules that grasp the BP and have antitumor activities via inhibition of splicing. There are many classes of these small molecules, termed splicing inhibitors/modulators, of which pladienolides, herboxidiene, and their analogues are well-studied examples^19,49–51^. A number of mutations in the HEAT repeats of SF3B1 have been reported that result in tolerance to splicing inhibitors^18,21,22,52,53^. In a previous study, we evolved a series of splicing-inhibitor tolerant SF3B1 versions in rice; these versions carry mutations in the HEAT repeats or the unstructured part of SF3B1^33,54,55^.

The SGR4 carries the substitutions in the HEAT repeat (K1049R, K1050E, and G1051H). The detailed phenotypic analysis of SGR4 carrying the *SF3B1* mutations at different developmental stages showed that these plants are insensitive to splicing inhibitors. These inhibitors affect germination and primary root growth. Lateral root growth is not only controlled by a different mechanism than primary root^56–58^ but also requires a functional splicing machinery. We showed here that lateral roots as well as the other phenotypes induced by splicing inhibitors were not observed in SGR4 lines. These observations are further supported by global transcriptomic and splicing analyses. Our analyses indicate that these SGR mutants are barely affected in transcription and splicing compared to the large impact observed by the treatment with splicing inhibitors to the WT.

### Expression of engineered PHF5A confers tolerance to splicing inhibitors

PHF5A is another protein of the SF3B complex and is involved in BP interaction together with SF3B1. Studies have shown that splicing inhibitors bind and fit in the pocket of the PHF5A–SF3B1 complex and consequently disrupt the interaction with BP. A single amino acid substitution in PHF5A (Y36C) interrupts the binding with splicing inhibitors and results in tolerance to these splicing inhibitors^18,22^. Our analyses also indicate that the overexpression of PHF5A-Y36C confers tolerance to splicing inhibition in rice. Rice has two identical homologs of human PHF5A, which were probably evolutionarily conserved to help the plant survive environmental challenges. We used CRISPR/Cas9 to target both loci simultaneously to produce a complete *OsPHF5A* knockout mutant in rice. Of note, we also used two sgRNAs to target the two *OsPHF5A* loci simultaneously in an attempt to generate a complete knockout of PHF5A function in plants, but we did not recover a homozygous double knockout mutant for both *OsPHF5A* loci. Recent structural analysis data indicate that PHF5A-Y36C forms a direct contact with BPA, thus implicating PHF5A function in BP recognition^18,30^. Such an essential role of PHF5A in splicing may explain our inability to recover a double mutant using CRISPR/Cas9-targeted mutagenesis; it appears that an *OsPHF5A* double mutant is embryonic lethal. Interestingly, we produced single knockout mutants for both of the *PHF5A* loci in rice. These single knockout mutants also showed a certain degree of tolerance to splicing inhibitors, albeit weaker than the one conferred by PHF5A-Y36C. We performed global gene expression and splicing analyses and found that under control conditions, the number of DEGs and DAS genes are significantly higher in PHF5A-Y36C-expressing plants compared to WT plants. This observation is in contrast to what is observed in human cell lines where expression of PHF5A-Y36C does not significantly affect splicing^18^.

In yeast, the spliceosome undergoes multiple ATP-dependent conformational changes. After activation of the spliceosome, Prp2 removes U2 component SF3a/b to allow the interaction of the BP and the 5′ SS so that the catalytic reaction can take place. The Prp2 activity is ATP-dependent and it moves in a 3′ to 5′ direction toward the branch site to displace SF3a/b from the spliceosome. The dissociation of SF3a/b is necessary for the binding of Cwc25 to facilitate the branching reaction^13,59–61^. Based on the yeast splicing mechanism, we speculate that in PHF5A-Y36C-expressing plants under normal conditions, a high amount of PHF5A is produced and this increases the likelihood that PHF5A remain bound to the U2 snRNP during the catalytic phase. This delays the release of SF3a/b from the spliceosome and causes partial disruption of pre-mRNA splicing. However, under GEX1A treatment, the WT PHF5A is not functional and only PHF5A-Y36C is able to pass through the activation and catalytic phase and subsequent splicing steps. Taken together, accurate control of PHF5A homeostasis is needed for precise pre-mRNA splicing reaction under normal or stress conditions.

### Regulation of gene expression and AS to fine-tune plant responses to abiotic stresses

Plants as sessile organisms have evolved complex structures and systems to cope with environmental stresses and adapt their growth and development. Rapid perception and consequent reprogramming of gene expression play a significant role in the adaptation of plants to abiotic stress and environmental constraints. The splicing process is accomplished by hundreds of proteins including the splicing factors (SFs) serine/arginine-rich (SR) proteins and heterogeneous nuclear ribonucleoproteins (hnRNPs). These SFs bind to the regulatory *cis*-elements in the pre-mRNA thus act as activators and repressors of splice site selection^62,63^. However, the pre-mRNA of these SFs can also be alternatively spliced under different environmental conditions emphasizing the occurrence of feedbacks in splicing regulation. Transcription factors (TFs) are the principal regulators of plant growth and development and stress-responsive TFs like AREB, DREB, NAC, and 14-3-3 also undergo alternative splicing under abiotic stress conditions^64–67^. Understanding the molecular basis of this regulation will unlock the potential to harness the splicing machinery to answer basic biological questions and for biotechnological applications. Small-molecule splicing inhibitors capable of targeting different splicing factors provide invaluable tools to underpin the molecular regulation of the splicing machinery^31,32,68^.

**Fig. 9:**
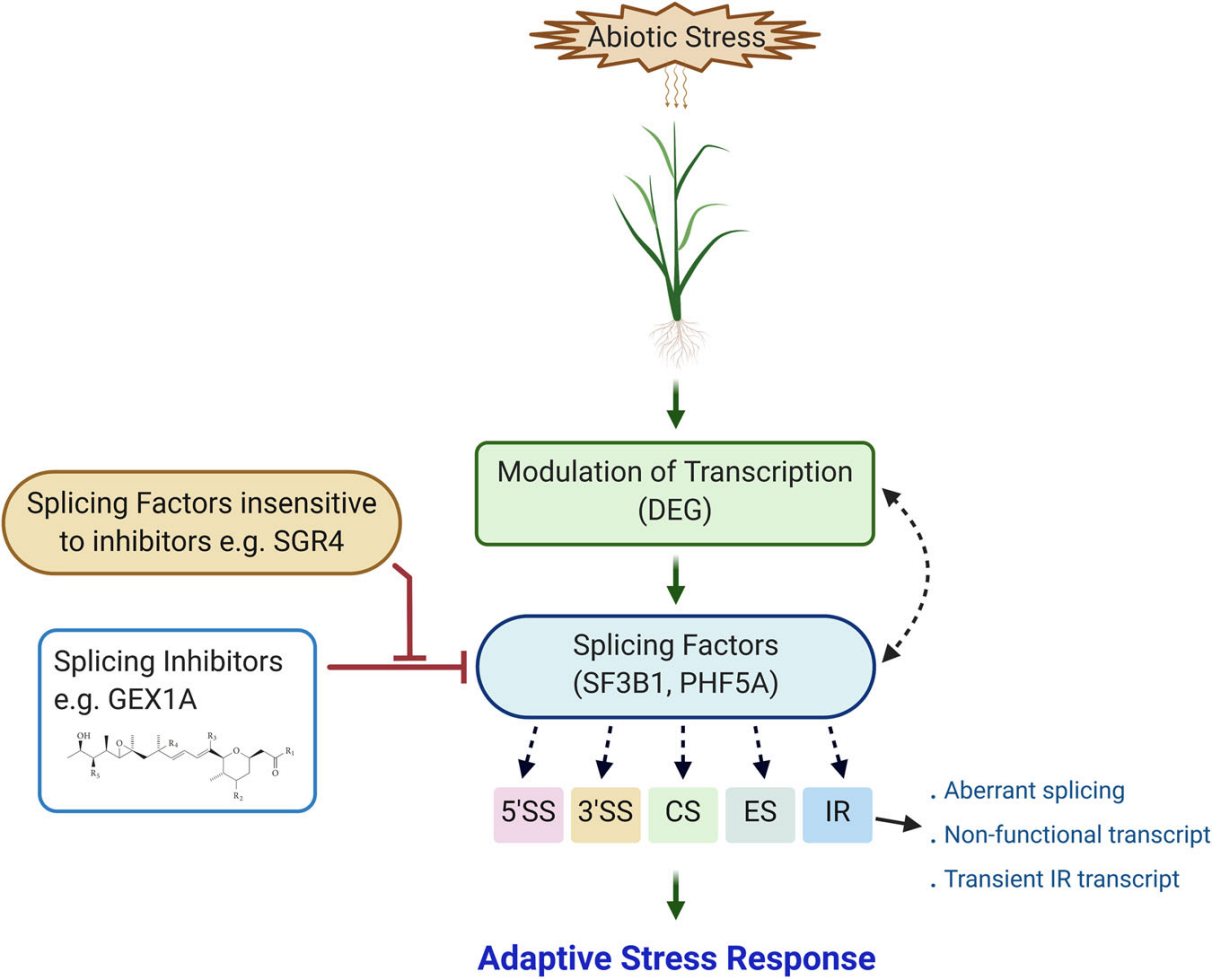
Model for splicing-mediated adaptive stress responses. Abiotic stress affects global gene expression by reprogramming it at the transcriptional and post-transcriptional levels. Plants perceive stress signals through multiple integrated signaling pathways, which regulate transcription and splicing processes. Transcription factors affect the expression of splicing factors, and in turn splicing factors regulate the splicing patterns of transcription factors. Splicing inhibitors (e.g., GEX1A) inhibit the splicing and activate the stress signaling in plants. The splicing factor protein variants insensitive to GEX1A (e.g., SGR4) impede the inhibitors’ effects of splicing inhibitors. Stress-modulated transcripts and splice isoforms help plants to cope with abiotic stresses. Arrows indicate activation or repression, and T-bars indicate repression. CS constitutive splicing, ES exon skipping, 5′ SS 5′ splice site, 3′ SS 3′ splice site, IR intron retention.

Our work also established a link between splicing inhibition and responses to abiotic stress. Based on our observations, we propose a model linking modulation of splicing factors action with adaptive stress responses (Fig. 9). When a plant submits an environmental stress there are several responses at transcriptional level but also at post-transcriptional levels such as AS. In mutants where splicing sensitivity to a particular drug is affected, the effect of stress is different than the one observed in wild-type pointing to an overlay regulatory level of AS acting during stress adaptation. Specific isoforms of transcription factors may have different roles on gene regulation and may also control the expression of splicing factors necessary for the induction of those isoform changes revealing a complex interaction between splicing regulation and TF action. Hence, TFs affect the expression of splicing factors and in turn, these splicing factors regulate their splicing patterns and indirectly their potential function. These regulatory networks involving TFs, SFs and their targets may help plants to cope with abiotic stresses (Fig. 9). From stress perception to adaptive stress response, the pathway is intertwined at many steps. This is further validated by previous work that indicates AS has regulatory functions during plant development^6,57,69–71^.

Our study sets a milestone for the use of splicing modulators to fine-tune the splicing mechanism to cope under abiotic stress conditions. Finally, there is a pressing need to develop cheaper analogues of splicing inhibitors to use as herbicides and our findings can have potential applications for generating crops with herbicide tolerance.

## Methods

### Plant materials, chemicals, and vector construction

*Oryza sativa* L. ssp. *japonica* cv Nipponbare was used for all experiments. The SGR4 mutant is described elsewhere^33^.

GEX1A/ Herboxidiene (CAS: 142861-00-5) was purchased from BOC Sciences (45-16 Ramsey Road, Shirley, NY 11967, USA).

To target *OsPHF5A (LOC_Os04g56760)* and *OsPHF5A-Like (LOC_Os05g30410)*, sgRNAs were cloned in the *pRGEB32* plasmid^72^. The expression of Cas9 was driven by *OsUbiquitin* and the sgRNA was expressed under the *OsU3* promoter. The *pRGEB32* plasmid was digested with *Bsa*I and sgRNAs were synthesized as oligonucleotides with *Bsa*I overhangs, GGCA in the forward and AAAC in the reverse oligonucleotides. The oligonucleotides were annealed and ligated in the *Bsa*I-digested vector. The oligonucleotide sequences are listed in Supplementary Table 1.

To generate the PHF5A-Y36C heterologous expression line, the synthetic fragment of rice *OsPHF5A (LOC_Os04g56760)* with Y36C modification and 3xFLAG-tag at N-terminus was cloned under the control of the *OsUbiquitin* promoter via a Gateway cloning strategy. The sequence of the synthetic fragment is given in Supplementary Table 1.

### Rice transformation and genotyping of transgenic plants

Rice transformation was performed using the agrobacterium strain EHA105 and genotyped as described previously^56^. Briefly, the sgRNA-targeted region was amplified by PCR using locus-specific primers. Purified PCR products were cloned using CloneJET PCR Cloning Kit (K1231). Sanger sequencing was conducted to analyze the mutation.

### Phenotypic analysis

The germination inhibition assays were performed in six-well culture plates. Rice seeds were surface-sterilized and germinated in 4 mL of H_2_O for controls and with 5 µM and 10 µM of GEX1A. The germination rate was calculated after 5 d.

For the primary root elongation inhibition assay, sterilized rice seeds were germinated on ½ MS agar media (without sucrose) square plates for three days in a vertical position. On the third day, seedlings with similar root lengths were transferred to ½ MS media (without sucrose) supplemented with different concentrations of GEX1A and/or NaCl and grown in a vertical position. The tip of the root was marked and subsequent root growth was calculated after 3 days.

The elongated root length after transfer to control plates was set at 1 (100%). The root elongation rates on the chemical‐containing plates were calculated as (elongated root length on chemical)/ (elongated root length on control) × 100%. Values are means ± SE (*n* = 3). Significance (*P* < 0.05) was assessed by the Student’s *t*‐test.

For lateral root inhibition analysis, rice seeds were germinated on ½ MS agar media (without sucrose) square plates for three days. Seedlings with similar root size were transferred to ½ MS media (without sucrose) supplemented with 0.5 µM and 1 µM GEX1A plates. Lateral root growth was observed 3 days after transfer using a Nikon SMZ25 stereomicroscope.

### Immunoblot analysis

Total proteins were extracted from 100 mg of sample using extraction buffer (100 mM Tris-Cl pH8, 150 mM NaCl, 0.6% IGEPAL, 1 mM EDTA, 3 mM DTT with protease inhibitors, PMSF, leupeptin, aprotinin, pepstatin, antipain, chymostatin, Na_2_VO_3_, NaF, MG132, and MG115. Proteins were separated on a 10% polyacrylamide gel. Immunoblot analysis was carried out using mouse α-FLAG M2 (dilution, 1:1000) antibody for FLAG-PHF5A from OGR transgenic rice plants. The antigens were detected by chemiluminescence using an ECL-detecting reagent (Thermo Scientific).

### RNA isolation and RNA‐seq

Total RNA was extracted from 7-day-old rice seedlings after 6 h of treatment with DMSO, 0.3 µM GEX1A or 200 mM NaCl using the Direct-zol RNA MiniPrep Plus (Zymo Research) according to the manufacturer’s instructions. RNA was quantified using a Nanodrop and RNA quality was examined using a 2100 Bioanalyzer (Agilent Technologies). High-quality RNA samples with RNA integrity number (RIN) ≥ 7.0 were selected for library construction. The RNA-seq libraries were constructed using TruSeq mRNA stranded kit following the standard protocol and sequenced on the HiSeq-4000 platform to generate high-quality paired-end reads.

### RT-PCR and RT-qPCR

For reverse-transcription PCR (RT-PCR), DNA digestion of total RNA samples was performed using an RNase-Free DNase Set (Invitrogen cat. No. 18068-015) following the manufacturer’s protocol. The total RNA was reverse transcribed using a SuperScript First-Strand Synthesis System (Invitrogen) to generate cDNA. PCR conditions were: initial denaturation at 95 °C for 2 min, then 40 cycles of 95 °C for 30 sec, 55 °C for 30 sec, and 72 °C for 60 sec, then final elongation at 72 °C for 5 min. Primers used for RT-PCR are listed in Supplementary Table 1.

The qPCR was performed using the 50 ng RNA in 10 µl final reaction volume. The iTaq^TM^ Universal SYBR® Green One-Step Kit (Bio-Rad, cat. no. 172-5150) was used with the following manufacturer’s protocol conditions: reverse transcription 50 °C for 10 min, followed by 40 cycles of 95 °C for 15 s, 60 °C for 1 min, melt-curve analysis 65–95 °C, 0.5 °C increment. Primers used for qPCR are listed in Supplementary Table 2.

### Analysis of RNA-seq data and gene functional classification

RNA-seq preprocessing included trimming library adapters and quality controls with Trimmomatic using the following arguments; TrimmomaticPE - LEADING:25 TRAILING:25 CROP:120 MINLEN:120. Illumina adapters were removed and read pairs were trimmed to the same length. Processed reads were aligned using STAR with the following arguments:–outSAMtype BAM SortedByCoordinate–outReadsUnmapped Fastx–readFilesCommand zcat,–quantMode GeneCounts,–outFilterMultimapNmax 1. Reads overlappings exons per genes were counted using the FeatureCounts function of the Rsubreads package using the previously published GTF annotation files^57^.

Significance of differential gene expression was estimated using DEseq2, and the FDR correction of the *p*-value was used during pairwise comparison between genotypes. A gene was declared differentially expressed if its adjusted *p*-value (FDR) was ≤ 0.01. Gene ontology enrichment analysis was done as in ref. ^58^ and simplified using GO slim terms.

### Analysis of differential splicing

Differential splicing was determined using rMATS v4.02 (http://rnaseq-mats.sourceforge.net/) using the following arguments -nthread 4 –readLength 120 -t paired –libType fr-firststrand. The gene annotation GTF file from^57^ was as a reference annotation. Events with FDR < 0.001 and |IncLevelDifference| >0.2 were defined as differentially alternatively spliced^21^.

### Clustering analysis

Comparison between sets of DEGs or DAS genes and events were done using the UpSetR and the eulerr R packages. Clustering was done using the hierarchical clustering function of the PheatMap package using euclidean distance. Clusters were extracted from the heatmap using the cutree function. From DEG, the log_2_ fold change (log_2_FC) values were used to cluster genes by their changes of expression. For splicing the IncLevelDifference of differentially retained introns was used to cluster splicing change profiles in the different genotypes.

### Intron and splice site analysis

The length and the GC content of sequences of differentially retained introns in SGR4 and PHF5 mutants were analyzed using custom scripts. SVM-BPfinder (regulatorygenomics.upf.edu/Software/SVM_BP/) was used for scoring BP and Py-tracts.

### Reporting summary

Further information on research design is available in the Nature Research Reporting Summary linked to this article.

## Supplementary information

Reporting Summary

Description of Additional Supplementary Files

Supplementary Information

Supplementary Data 1

Supplementary Data 2

Supplementary Data 3

Supplementary Data 4

Supplementary Data 5

Supplementary Data 6

Supplementary Data 7

Supplementary Data 8

Supplementary Data 9

Supplementary Data 10

## Data Availability

(1) The Raw sequencing data are available at SRA under the BioProject accession PRJNA636200. (2) The processed data (https://www.bam.fwd.bigWig) files are deposited in GEO (https://www.ncbi.nlm.nih.gov/geo/) under accession number GSE153234. The following secure token has been created to allow review of record GSE153234 while it remains in private status: ezgpgmkkdfyhzuv. Please note the following points: (i) This token allows anonymous, read-only access to GSE153234, and associated accessions while they are private. (ii) Treat the token as you would a password and realize that the token provides access to GSE153234 to anyone who uses it. (3) The lists of DEGs and DAS events were attached with the manuscript as supplementary data files.

## References

[1] Will C. L. & Lührmann R. Spliceosome structure and function. *Cold Spring Harb Perspect. Biol. ***3**, a003707 (2011). 10.1101/cshperspect.a003707 PMC3119917 21441581

[2] Plaschka, C., Pei-Chun, L. & Nagai, K. Structure of a pre-catalytic spliceosome. *Nature* **546**, 617 (2017). 10.1038/nature22799 PMC5503131 28530653

[3] MacMillan AM (1994). Dynamic association of proteins with the pre-mRNA branch region. Genes Dev..

[4] Wahl MC, Will CL, Luhrmann R (2009). The spliceosome: design principles of a dynamic RNP machine. Cell.

[5] Shi Y (2017). Mechanistic insights into precursor messenger RNA splicing by the spliceosome. Nat. Rev. Mol. Cell Biol..

[6] Staiger D, Brown JW (2013). Alternative splicing at the intersection of biological timing, development, and stress responses. Plant Cell.

[7] DeBoever C (2015). Transcriptome sequencing reveals potential mechanism of cryptic 3′ splice site selection in SF3B1-mutated cancers. PLoS Comput. Biol.

[8] Cretu C (2016). Molecular architecture of SF3b and structural consequences of its cancer-related mutations. Mol. Cell.

[9] Lee, B. M. & Christopher, L. A genomic view of alternative splicing. *Nat. Genet.* **30**, 13 (2002). 10.1038/ng0102-13 11753382

[10] Graveley TWN, Brenton R (2010). Expansion of the eukaryotic proteome by alternative splicing. Nature.

[11] Filichkin S, Priest HD, Megraw M, Mockler TC (2015). Alternative splicing in plants: directing traffic at the crossroads of adaptation and environmental stress. Curr. Opin. Plant Biol..

[12] Golas MM, Sander B, Will CL, Luhrmann R, Stark H (2003). Molecular architecture of the multiprotein splicing factor SF3b. Science.

[13] Lardelli RM, Thompson JX, Yates JR, Stevens SW (2010). Release of SF3 from the intron branchpoint activates the first step of pre-mRNA splicing. RNA.

[14] Hasegawa M (2011). Identification of SAP155 as the target of GEX1A (Herboxidiene), an antitumor natural product. ACS Chem. Biol..

[15] Kaida D (2007). Spliceostatin A targets SF3b and inhibits both splicing and nuclear retention of pre-mRNA. Nat. Chem. Biol..

[16] Malcovati L (2011). Clinical significance of SF3B1 mutations in myelodysplastic syndromes and myelodysplastic/myeloproliferative neoplasms. Blood.

[17] Kotake Y (2007). Splicing factor SF3b as a target of the antitumor natural product pladienolide. Nat. Chem. Biol..

[18] Teng T (2017). Splicing modulators act at the branch point adenosine binding pocket defined by the PHF5A-SF3b complex. Nat. Commun..

[19] Cretu C (2018). Structural basis of splicing modulation by antitumor macrolide compounds. Mol. Cell.

[20] Hansen SR, Nikolai BJ, Spreacker PJ, Carrocci TJ, Hoskins AA (2019). Chemical inhibition of pre-mRNA splicing in living Saccharomyces cerevisiae.. Cell Chem. Biol..

[21] Yokoi A (2011). Biological validation that SF3b is a target of the antitumor macrolide pladienolide. FEBS J..

[22] Finci LI (2018). The cryo-EM structure of the SF3b spliceosome complex bound to a splicing modulator reveals a pre-mRNA substrate competitive mechanism of action. Genes Dev..

[23] Gozani, O., Potashkin, J. & Reed, R. A potential role for U2AF-SAP 155 interactions in recruiting U2 snRNP to the branch site. *Mol. Cell Biol.* **18**, 4752–4760 (1998). 10.1128/mcb.18.8.4752 PMC109061 9671485

[24] Cass DM, Berglund JA (2006). The SF3b155 N-terminal domain is a scaffold important for splicing. Biochemistry.

[25] Thickman KR, Swenson MC, Kabogo JM, Gryczynski Z, Kielkopf CL (2006). Multiple U2AF65 binding sites within SF3b155: thermodynamic and spectroscopic characterization of protein-protein interactions among pre-mRNA splicing factors. J. Mol. Biol..

[26] Trappe R (2002). Identification and characterization of a novel murine multigene family containing a PHD-finger-like motif. Biochem. Biophys. Res. Commun..

[27] Strikoudis A (2016). Regulation of transcriptional elongation in pluripotency and cell differentiation by the PHD-finger protein Phf5a. Nat. Cell Biol..

[28] Yang Y (2018). PHD-finger domain protein 5A functions as a novel oncoprotein in lung adenocarcinoma. J. Exp. Clin. Cancer Res.

[29] Zheng YZ (2018). PHF5A epigenetically inhibits apoptosis to promote breast cancer progression. Cancer Res.

[30] Yang Q (2019). Knockdown of PHF5A inhibits migration and invasion of HCC cells via downregulating NF-kappaB signaling. Biomed. Res. Int..

[31] AlShareef S (2017). Herboxidiene triggers splicing repression and abiotic stress responses in plants. BMC Genomics.

[32] Ling Y (2017). Pre-mRNA splicing repression triggers abiotic stress signaling in plants. Plant J..

[33] Butt H (2019). CRISPR directed evolution of the spliceosome for resistance to splicing inhibitors. Genome Biol.

[34] Sun, C. The SF3b complex: splicing and beyond. *Cell Mol. Life Sci*. **77**, 3583–3595 (2020). 10.1007/s00018-020-03493-z PMC7452928 32140746

[35] Schellenberg MJ, Dul EL, MacMillan AM (2011). Structural model of the p14/SF3b155. branch duplex complex. RNA.

[36] Bonnal S, Vigevani L, Valcarcel J (2012). The spliceosome as a target of novel antitumour drugs. Nat Rev. Drug Discov..

[37] Amit M (2012). Differential GC content between exons and introns establishes distinct strategies of splice-site recognition. Cell Rep..

[38] Vigevani L, Gohr A, Webb T, Irimia M, Valcarcel J (2017). Molecular basis of differential 3’ splice site sensitivity to anti-tumor drugs targeting U2 snRNP. Nat. Commun..

[39] Feng J (2015). SKIP confers osmotic tolerance during salt stress by controlling alternative gene splicing in Arabidopsis. Mol. Plant.

[40] Zhan X (2015). An Arabidopsis PWI and RRM motif-containing protein is critical for pre-mRNA splicing and ABA responses. Nat. Commun..

[41] Thatcher SR (2016). Genome-wide analysis of alternative splicing during development and drought stress in maize. Plant Physiol.

[42] Jiang J (2017). Integrating omics and alternative splicing reveals insights into grape response to high temperature. Plant Physiol..

[43] Carrasco-Lopez C (2017). Environment-dependent regulation of spliceosome activity by the LSM2-8 complex in Arabidopsis. Nucleic Acids Res..

[44] Esteve-Bruna D (2020). Prefoldins contribute to maintaining the levels of the spliceosome LSM2-8 complex through Hsp90 in Arabidopsis. Nucleic Acids Res..

[45] Albaqami M, Laluk K, Reddy ASN (2019). The Arabidopsis splicing regulator SR45 confers salt tolerance in a splice isoform-dependent manner. Plant Mol. Biol.

[46] Corrionero A, Minana B, Valcarcel J (2011). Reduced fidelity of branch point recognition and alternative splicing induced by the anti-tumor drug spliceostatin A. Genes Dev.

[47] Bessonov S (2010). Characterization of purified human Bact spliceosomal complexes reveals compositional and morphological changes during spliceosome activation and first step catalysis. RNA.

[48] Rauhut R (2016). Molecular architecture of the Saccharomyces cerevisiae activated spliceosome. Science.

[49] Pham D, Koide K (2016). Discoveries, target identifications, and biological applications of natural products that inhibit splicing factor 3B subunit 1. Nat. Prod. Rep..

[50] Effenberger KA, Urabe VK, Jurica MS (2017). Modulating splicing with small molecular inhibitors of the spliceosome. Wiley Interdiscip. Rev. RNA.

[51] DeNicola AB, Tang Y (2019). Therapeutic approaches to treat human spliceosomal diseases. Curr. Opin. Biotechnol..

[52] Jin S (2017). Splicing factor SF3B1K700E mutant dysregulates erythroid differentiation via aberrant alternative splicing of transcription factor TAL1. PLoS ONE.

[53] Serrat X (2019). CRISPR editing of sftb-1/SF3B1 in Caenorhabditis elegans allows the identification of synthetic interactions with cancer-related mutations and the chemical inhibition of splicing. PLoS Genet..

[54] Butt, H., Zaidi, S. S., Hassan, N. & Mahfouz, M. CRISPR-based directed evolution for crop improvement. *Trends Biotechnol*. **38,** 236–240 (2019). 10.1016/j.tibtech.2019.08.001 31477243

[55] Zhang Y, Qi Y (2019). CRISPR enables directed evolution in plants. Genome Biol.

[56] Butt, H., Piatek, A., Li, L., Reddy, A. S. N. & Mahfouz, M. M. Multiplex CRISPR mutagenesis of the serine/arginine-rich (SR) gene family in rice. *Genes (Basel)* **10**, 596 (2019). 10.3390/genes10080596 PMC6723545 31394891

[57] Dong C (2018). Alternative splicing plays a critical role in maintaining mineral nutrient homeostasis in rice (Oryza sativa). Plant Cell.

[58] Horan K (2008). Annotating genes of known and unknown function by large-scale coexpression analysis. Plant Physiol.

[59] Warkocki Z (2009). Reconstitution of both steps of Saccharomyces cerevisiae splicing with purified spliceosomal components. Nat. Struct. Mol. Biol..

[60] Liu HL, Cheng SC (2012). The interaction of Prp2 with a defined region of the intron is required for the first splicing reaction. Mol. Cell Biol..

[61] Liu YC, Cheng SC (2015). Functional roles of DExD/H-box RNA helicases in pre-mRNA splicing. J. Biomed. Sci..

[62] Yeap, W. C., Namasivayam, P. & Ho, C. L. HnRNP-like proteins as post-transcriptional regulators. *Plant Sci*. **227**, 90–100 (2014). 10.1016/j.plantsci.2014.07.005 25219311

[63] Howard, J. M. & Sanford, J. R. The RNAissance family: SR proteins as multifaceted regulators of gene expression. *Wiley Interdiscip. Rev. RNA*** 6,** 93–110 (2015). 10.1002/wrna.1260 PMC4268343 25155147

[64] Lata, C. & Prasad, M. Role of DREBs in regulation of abiotic stress responses in plants. *J. Exp. Bot*. **62,** 4731–4748 (2011). 10.1093/jxb/err210 21737415

[65] Todaka, D., Nakashima, K., Shinozaki, K. & Yamaguchi-Shinozaki, K. Toward understanding transcriptional regulatory networks in abiotic stress responses and tolerance in rice. *Rice (NY)* **5,** 6 (2012). 10.1186/1939-8433-5-6 PMC3834508 24764506

[66] Nakashima, K., Ito, Y. & Yamaguchi-Shinozaki, K. Transcriptional regulatory networks in response to abiotic stresses in Arabidopsis and grasses. *Plant Physiol*. **149,** 88–95 (2009). 10.1104/pp.108.129791 PMC2613698 19126699

[67] Filichkin, S. A. et al. Abiotic Stresses Modulate Landscape of Poplar Transcriptome via Alternative Splicing, Differential Intron Retention, and Isoform Ratio Switching. *Front. Plant Sci.* **9,** 5 (2018). 10.3389/fpls.2018.00005 PMC5816337 29483921

[68] Lee, S. C. W. & Abdel-Wahab, O. Therapeutic Targeting of Splicing in Cancer. *Nat. Med.* **22,** 976–986 (2016). 10.1038/nm.4165 PMC5644489 27603132

[69] Shen, Y. et al. Global dissection of alternative splicing in paleopolyploid soybean. *Plant Cell* **26,** 996–1008 (2014). 10.1105/tpc.114.122739 PMC4001406 24681622

[70] Chaudhary, S., Jabre, I., Reddy, A. S. N., Staiger, D. & Syed, N. H. Perspective on Alternative Splicing and Proteome Complexity in Plants. *Trends. Plant. Sci.* **24,** 496–506 (2019). 10.1016/j.tplants.2019.02.006 30852095

[71] Xin, R., Kathare, P. K. & Huq, E. Coordinated Regulation of Pre-mRNA Splicing by the SFPS-RRC1 Complex to Promote Photomorphogenesis. *Plant Cell* **31,** 2052–2069 (2019). 10.1105/tpc.18.00786 PMC6751115 31266850

[72] Xie K, Minkenberg B, Yang Y (2015). Boosting CRISPR/Cas9 multiplex editing capability with the endogenous tRNA-processing system. Proc. Natl Acad. Sci. USA.

