# Supplementary material for: Overlapping roles of spliceosomal components SF3B1 and PHF5A in rice splicing regulation
Source: Commun Biol. 2021 May 5;4:529. doi: 10.1038/s42003-021-02051-y (PMC8100303; doi:10.1038/s42003-021-02051-y)
Supplement: Supplementary file 1 — Reporting Summary [file 42003_2021_2051_MOESM1_ESM.pdf]

nature research

Corresponding author(s): *Double-blind peer review submissions: write DBPR and your manuscript number here instead of author names.*

Last updated by author(s): Mar 5, 2021

Reporting Summary

Nature Research wishes to improve the reproducibility of the work that we publish. This form provides structure for consistency and transparency in reporting. For further information on Nature Research policies, see our Editorial Policies and the Editorial Policy Checklist.

Statistics

For all statistical analyses, confirm that the following items are present in the figure legend, table legend, main text, or Methods section.

n/a	Confirmed	
☐	☒	The exact sample size (*n*) for each experimental group/condition, given as a discrete number and unit of measurement
☐	☒	A statement on whether measurements were taken from distinct samples or whether the same sample was measured repeatedly
☐	☒	The statistical test(s) used AND whether they are one- or two-sided<br>*Only common tests should be described solely by name; describe more complex techniques in the Methods section.*
☐	☒	A description of all covariates tested
☐	☒	A description of any assumptions or corrections, such as tests of normality and adjustment for multiple comparisons
☐	☒	A full description of the statistical parameters including central tendency (e.g. means) or other basic estimates (e.g. regression coefficient) AND variation (e.g. standard deviation) or associated estimates of uncertainty (e.g. confidence intervals)
☐	☒	For null hypothesis testing, the test statistic (e.g. *F*, *t*, *r*) with confidence intervals, effect sizes, degrees of freedom and *P* value noted<br>*Give P values as exact values whenever suitable.*
☒	☐	For Bayesian analysis, information on the choice of priors and Markov chain Monte Carlo settings
☐	☒	For hierarchical and complex designs, identification of the appropriate level for tests and full reporting of outcomes
☒	☐	Estimates of effect sizes (e.g. Cohen's *d*, Pearson's *r*), indicating how they were calculated

Our web collection on statistics for biologists contains articles on many of the points above.

Software and code

Policy information about availability of computer code

Data collection	The plant images are taken with Nikon camera. Lateral root growth was observed using a Nikon SMZ25 stereomicroscope. The RNA-seq libraries sequenced on the HiSeq-4000 platform.
Data analysis	All software and code used for data analysis are listed in the methods section.

For manuscripts utilizing custom algorithms or software that are central to the research but not yet described in published literature, software must be made available to editors and reviewers. We strongly encourage code deposition in a community repository (e.g. GitHub). See the Nature Research guidelines for submitting code & software for further information.

Data

Policy information about availability of data

All manuscripts must include a data availability statement. This statement should provide the following information, where applicable:

- Accession codes, unique identifiers, or web links for publicly available datasets
- A list of figures that have associated raw data
- A description of any restrictions on data availability

1. The Raw sequencing data are available at SRA under the BioProject accession PRJNA636200. (https://dataview.ncbi.nlm.nih.gov/object/PRJNA636200?reviewer=oj95acuu9u231o8bcnn13ciocq)

2. The processed datasets (.bam.fwd.bigWig) that support the findings of this study are available in GEO (https://www.ncbi.nlm.nih.gov/geo/) under accession number GSE153234. The following secure token has been created to allow review of record GSE153234 while it remains in private status: ezgpgmkkdfyhzuv.

Field-specific reporting

Please select the one below that is the best fit for your research. If you are not sure, read the appropriate sections before making your selection.

- [x] Life sciences
- [ ] Behavioural & social sciences
- [ ] Ecological, evolutionary & environmental sciences

For a reference copy of the document with all sections, see nature.com/documents/nr-reporting-summary-flat.pdf

Life sciences study design

All studies must disclose on these points even when the disclosure is negative.

Sample size	All sample sizes are reported in figure legends, Methods and source data. No sample size calculations were performed. The sample size was chosen based on the simultaneous processing and taking into account the samples size commonly chosen by similar studies by other research group. The respective experiments were repeated with the same sample size at least two times for reproducibility.
Data exclusions	No data were excluded from any analyses.
Replication	The conclusions and experimental findings reported in this study are based on results that could be successfully reproduced at least two times.
Randomization	All experiments were randomly allocated to the experimental. Measurements and sampling of plant tissues were performed by randomly selecting plants grown under the same conditions.
Blinding	The investigators were not blinded to group allocation during sample collection or analysis, as the information on the genotypes of materials was essential for the experiment design and analysis.

Reporting for specific materials, systems and methods

We require information from authors about some types of materials, experimental systems and methods used in many studies. Here, indicate whether each material, system or method listed is relevant to your study. If you are not sure if a list item applies to your research, read the appropriate section before selecting a response.

Materials & experimental systems

n/a	Involved in the study	
[ ]	[x]	Antibodies
[x]	[ ]	Eukaryotic cell lines
[x]	[ ]	Palaeontology and archaeology
[x]	[ ]	Animals and other organisms
[x]	[ ]	Human research participants
[x]	[ ]	Clinical data
[x]	[ ]	Dual use research of concern

Methods

n/a	Involved in the study	
[x]	[ ]	ChIP-seq
[x]	[ ]	Flow cytometry
[x]	[ ]	MRI-based neuroimaging

Antibodies

Antibodies used	An anti-FLAG M2 antibody produced in mouse (Sigma; F3165) was used for WB (dilution, 1:1000). Secondary antibody anti-mouse (Sigma) was used for the detection of anti-FLAG antibody (dilution, 1:1000).
Validation	An antibody against FLAG was validated by detecting recombinant protein FLAG-PHF5A from transgenic rice plant leaves.
