# Supplementary material for: Overlapping roles of spliceosomal components SF3B1 and PHF5A in rice splicing regulation
Source: Commun Biol. 2021 May 5;4:529. doi: 10.1038/s42003-021-02051-y (PMC8100303; doi:10.1038/s42003-021-02051-y)
Supplement: Supplementary file 2 — Description of Additional Supplementary Files [file 42003_2021_2051_MOESM2_ESM.pdf]

Description of Additional Supplementary Data Files

File name: Supplementary Data 1.

Description:

WTX_vs_WTC. DEGs in WT after GEX1A treatment, SF3B1 experiment

SGR4X_vs_SGR4C. DEGs in SGR4 after GEX1A treatment, SF3B1 experiment

SGR4C_vs_WTC. DEGs after comparing SGR4 and WT under control conditions

SGR4X_vs_WTX. DEGs after comparing SGR4 and WT under GEX1A treatment

File name: Supplementary Data 2.

Description:

WTX_vs_WTC. DAS events in WT after GEX1A treatment, SF3B1 experiment

SGR4X_vs_SGR4C. DAS events in SGR4 after GEX1A treatment, SF3B1 experiment

SGR4C_vs_WTC. DAS events after comparing SGR4 and WT under control conditions

SGR4X_vs_WTX. DAS events after comparing SGR4 and WT under GEX1A treatment

File name: Supplementary Data 3.

Description:

WTX_vs_WTC. DEGs in WT after GEX1A treatment, PHF5A experiment

OGRX_vs_OGRC. DEGs in SGR after GEX1A treatment, PHF5A experiment

OGRC_vs_WTC. DEGs after comparing OGR and WT under control conditions

OGRX_vs_WTX. DEGs after comparing OGR and WT under GEX1A treatment

File name: Supplementary Data 4.

Description:

WTX_vs_WTC. DAS events in WT after GEX1A treatment, PHF5A experiment

OGRX_vs_OGRC. DAS events in OGR after GEX1A treatment, PHF5A experiment

OGRC_vs_WTC. DAS events after comparing OGR and WT under control conditions

OGRX_vs_WTX. DAS events after comparing OGR and WT under GEX1A treatment

File name: Supplementary Data 5.

Description:

Salt_DEG. DEGs in 7-day-old WT seedlings after 200 mM NaCl treatment for 6 h

Salt_DAS. DAS events in 7-day-old WT seedlings after 200 mM NaCl treatment for 6 h

File name: Supplementary Data 6

Description:

Data point Figure_1C.

File name: Supplementary Data 7

Description:

Data point Figure_1E.

File name: Supplementary Data 8

Description:

Data point Figure_3F.

File name: Supplementary Data 9

Description:

Data point Figure_7B.

File name: Supplementary Data 10

Description:

Data point Figure_8A.
