# Supplementary material for: Overlapping roles of spliceosomal components SF3B1 and PHF5A in rice splicing regulation
Source: Commun Biol. 2021 May 5;4:529. doi: 10.1038/s42003-021-02051-y (PMC8100303; doi:10.1038/s42003-021-02051-y)
Supplement: Supplementary file 3 — Supplementary Information [file 42003_2021_2051_MOESM3_ESM.pdf]

Supplementary Information

Overlapping roles of spliceosomal components SF3B1 and PHF5A in rice splicing regulation

Haroon Butt[1†], Jeremie Bazin[2†], Sahar Alshareef[1], Ayman Eid[1], Moussa Benhamed[2], Anireddy S.N. Reddy[3], Martin Crespi[2], and Magdy M. Mahfouz[1,*]

[1]Laboratory for Genome Engineering and Synthetic Biology, King Abdullah University of Science and Technology (KAUST), Thuwal, Saudi Arabia.

[2]CNRS, INRA, Institute of Plant Sciences Paris-Saclay IPS2, Univ Paris Sud, Univ Evry, Univ Paris-Diderot, Sorbonne Paris-Cite, Universite Paris-Saclay, Orsay, France.

[3]Department of Biology and Program in Cell and Molecular Biology, Colorado State University, Fort Collins, CO 80523, USA

† These authors contributed equally

[*]Correspondence to Magdy Mahfouz (Magdy.mahfouz@kaust.edu.sa)

Key words: Spliceosome, SF3B complex, SF3B1, splicing modulators, herboxidiene, pladienolide B, spliceostatin A, CRISPR/Cas9, Directed evolution, genome engineering.

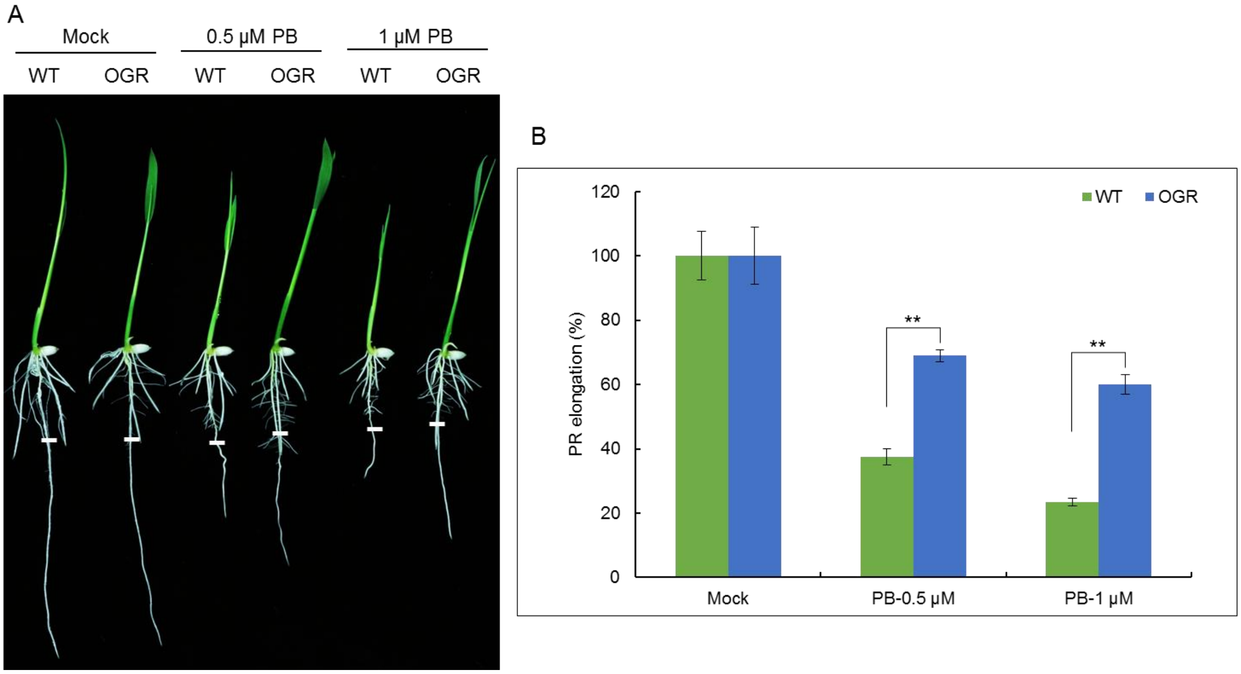

Supplementary Figure 1: Primary root growth of *OGR* shows resistance to PB treatment

(A, B) Rice seedlings were germinated on ½ MS basal salt media plates for 3 days in vertical position. Seedlings of similar root size were transferred to ½ MS media plates supplemented with 0 μM PB (mock), 0.5 μM PB, and 1 μM PB (n=6).

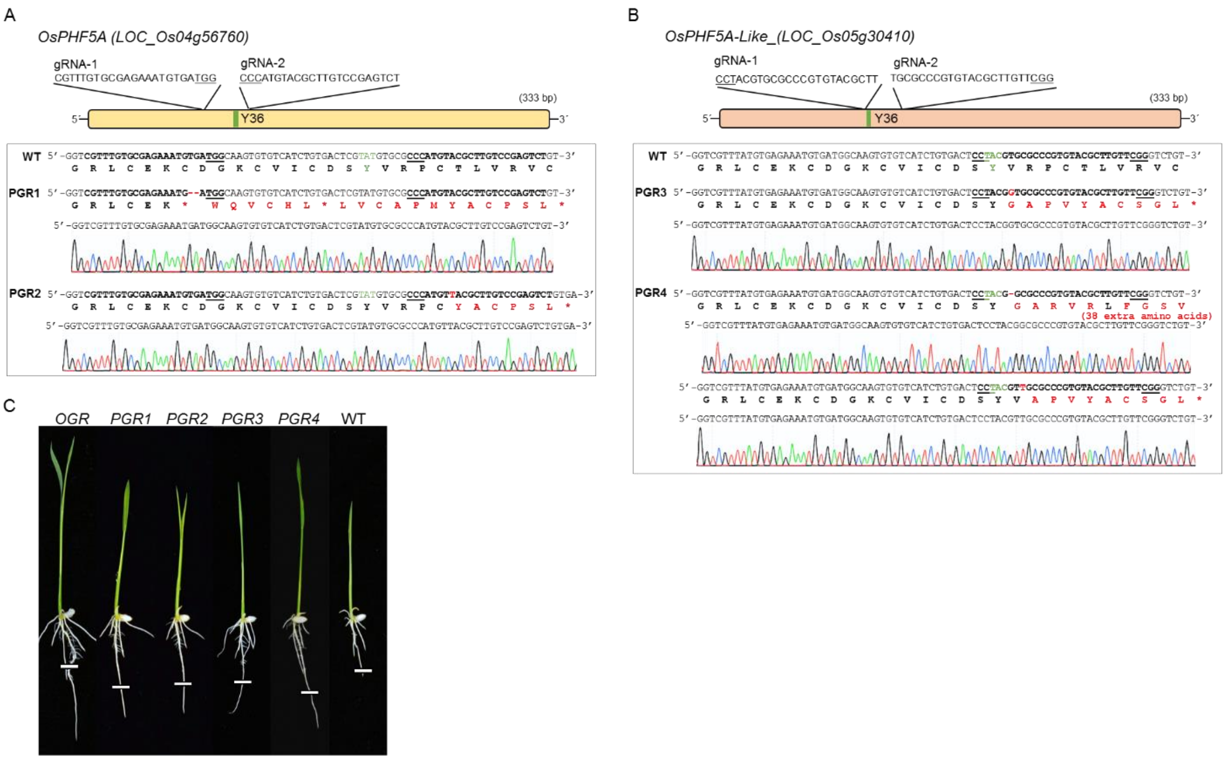

Supplementary Figure 2: CRISPR-targeted mutagenesis of splicing factor PHF5A in rice

(A, B) Rice has two loci for PHF5A. sgRNAs were designed to target the Y36 residue using the closest available PAM sequences. The mutants were termed PGR (PHF5A GEX1A Resistance). PGR1, PGR2, and PGR3 are monoallelic homozygous knock-out mutants whereas PGR4 is a biallelic mutant. We attempted to produce a double knock-out mutant targeting both genes but were not successful. **(C)** PGRs show tolerance to GEX1A treatment. Seeds were germinated on ½ MS media for three days. Seedlings were then transferred to ½ MS media supplemented with 0.3 µM Gex1A for three days. Root tips were marked to observe post-transfer growth.

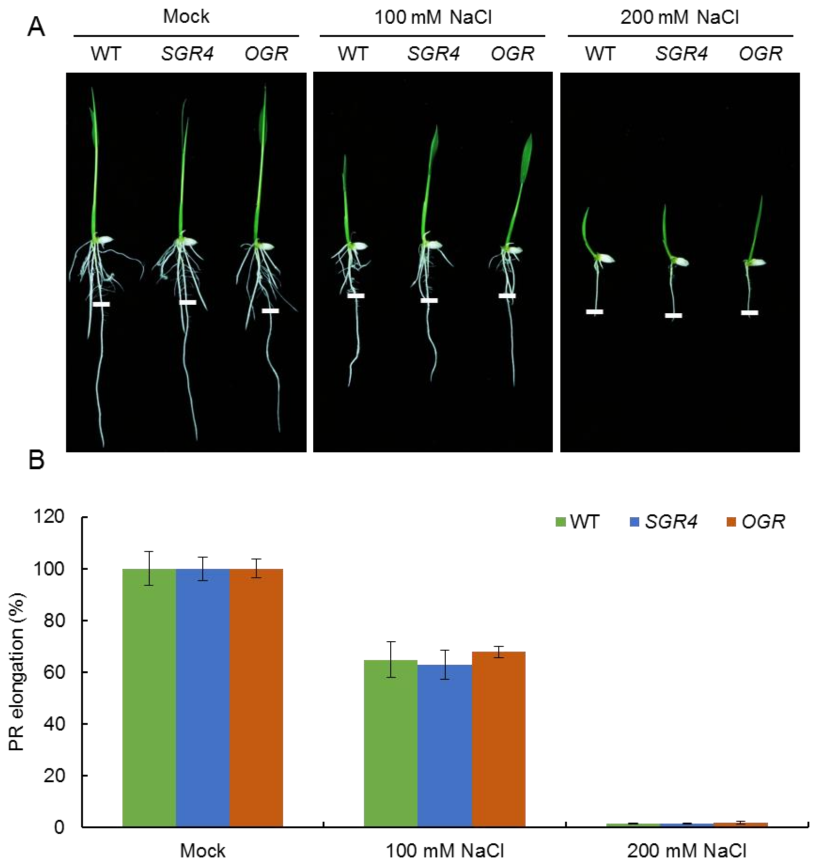

Supplementary Figure 3: Primary root growth of *OGR* and *SGR4* at different concentration of salt. (A, B) Rice seedlings were germinated on ½ MS basal salt media plates for 3 days in vertical position. Seedlings of similar root size were transferred to ½ MS media plates supplemented with 0 mM NaCl (mock), 100 mM NaCl, and 200 mM NaCl (n=6).

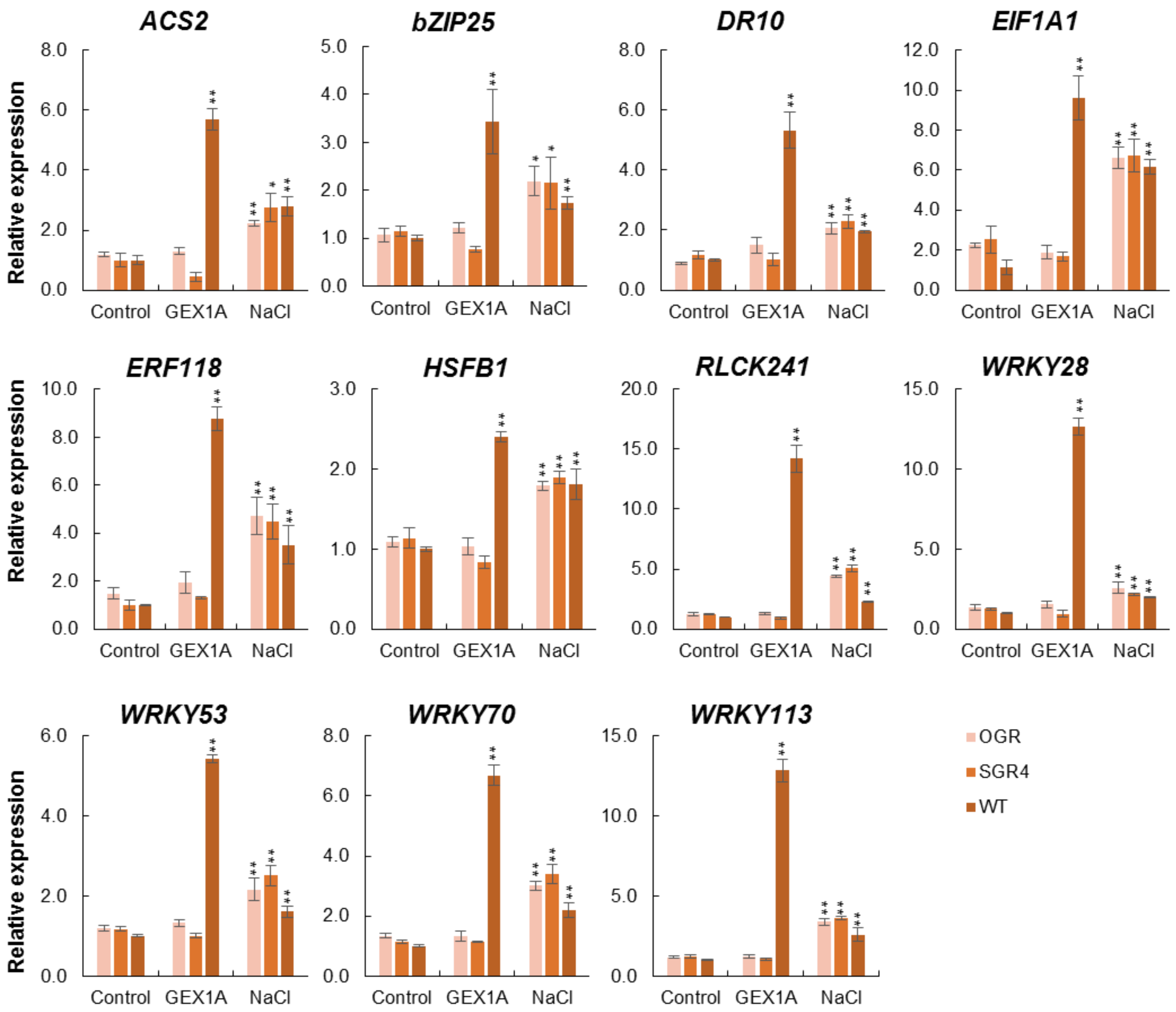

Supplementary Figure 4: Expression analysis of stress-responsive genes after GEX1A and salt treatment in OGR and SGR4.

Expression of stress-responsive genes in OGR, SGR4 and WT. The GEX1A triggers the expression patterns similar to salt stress treatment for all genes in WT but not in SGR4 and OGR. Bars represent mean ± SEM of three replicates. *OsActin* was used as an internal control. (Student's t-test; $^*P < 0.05$, $^{**}P < 0.01$).

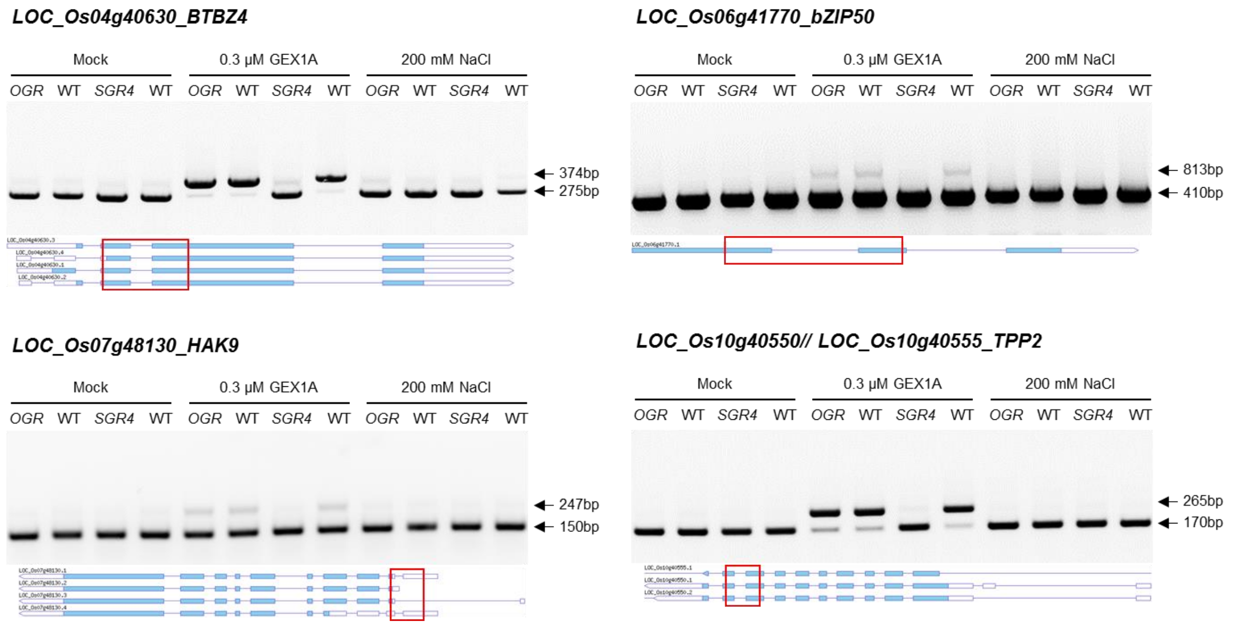

Supplementary Figure 5: Differential splicing pattern of stress-responsive genes after GEX1A and salt treatment in OGR and SGR4.

Semiquantitative RT-PCR analysis of alternative splicing patterns of stress-responsive genes in OGR, SGR4 and WT. No intron retention is observed in SGR4 under GEX1A treatment. Arrowheads indicate splicing variants that changed following GEX1A treatment. The gene structures and retained introns are shown. Red boxes indicate the PCR fragments.

*LOC_Os04g55970*_AP2-like

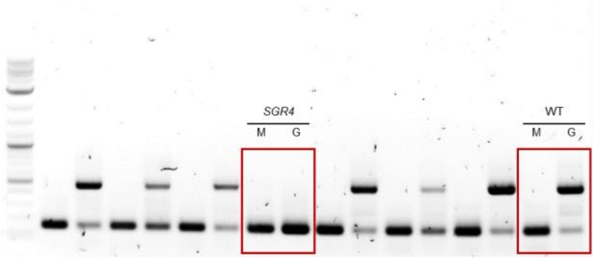

*LOC_Os07g42960*_phospho-2-dehydro-3-deoxyheptonate aldolase

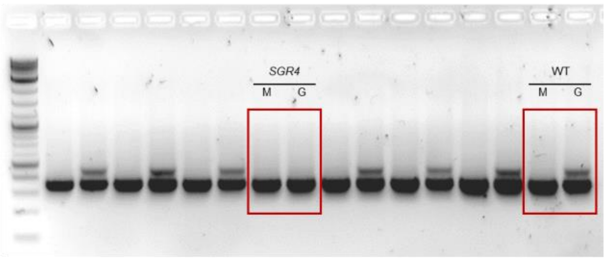

*LOC_Os04g57440*_oryzain beta chain precursor

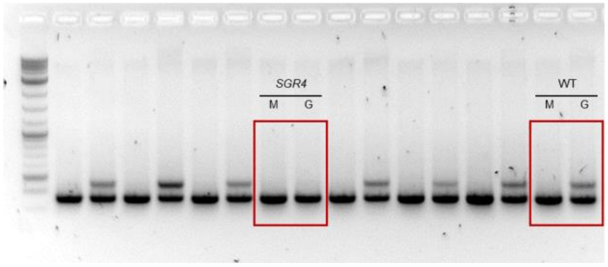

*LOC_Os03g15050*_PEP carboxykinase

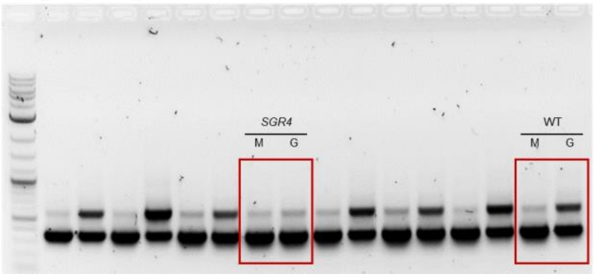

Supplementary Figure 6: Full gel images for Figure 1g.

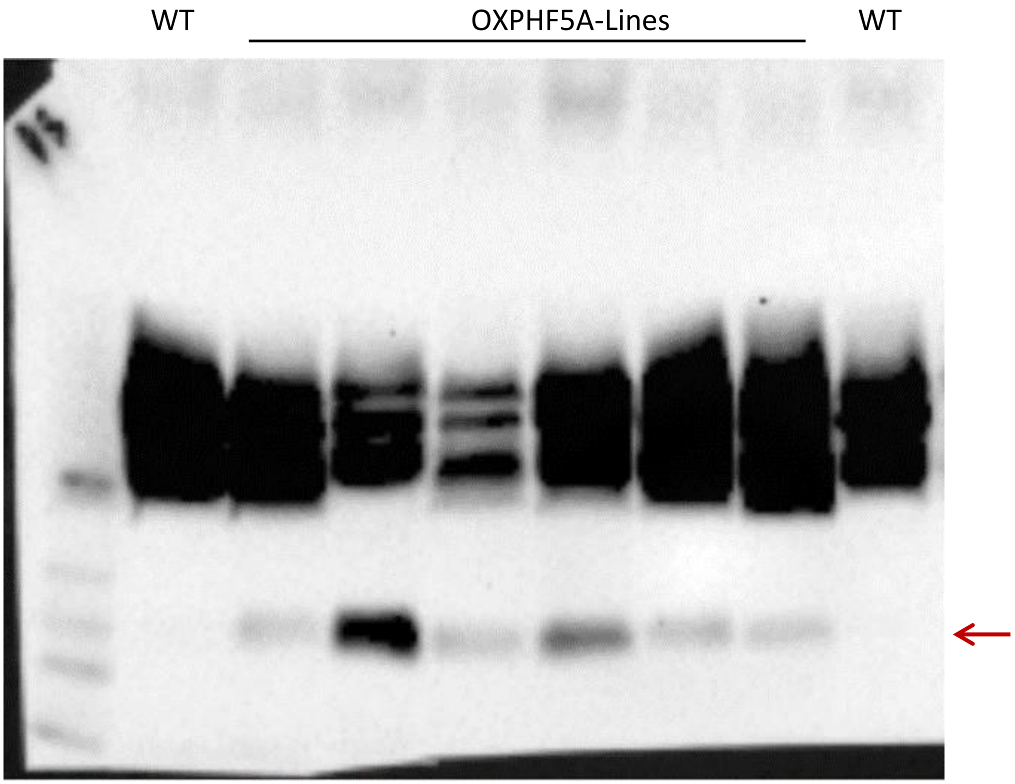

Supplementary Figure 7: Full Western blot for Figure 3d.

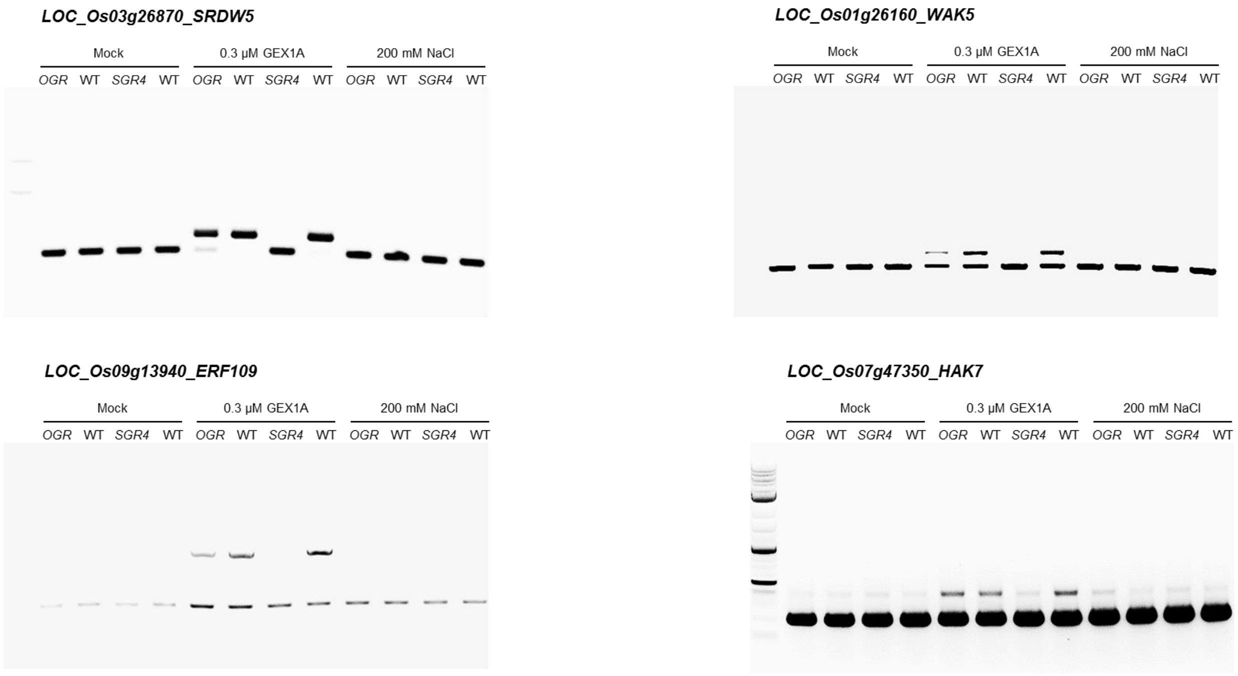

Supplementary Figure 8: Full gel images for Figure 8b.

Supplementary Table 1: List of sequences used in this study

Name of the primer	Sequence (5' - 3')	Purpose
07g42960_F	CGAGAGCTTCAAGGAGTTCAACG	SGR4 semi-quantitative RT-PCR
07g42960_R	ATGAAATCGAGGTTCCACTGCG	SGR4 semi-quantitative RT-PCR
03g15050_F	GTGTTTGTGAACGACCAGTTCCTG	SGR4 semi-quantitative RT-PCR
03g15050_R	CCTGCTTGCCCATGTTGCAG	SGR4 semi-quantitative RT-PCR
04g57440_F	CTGGGCCTTCTCCGCAGTAAG	SGR4 semi-quantitative RT-PCR
04g57440_R	ATGATAGAGCTGGAACTCGCGG	SGR4 semi-quantitative RT-PCR
04g55970_F	GAAGCATATGACACGCCAGGAG	SGR4 semi-quantitative RT-PCR
04g55970_R	GAATGTTCCGAGGTAGAGATCCTTG	SGR4 semi-quantitative RT-PCR
BTBZ4_F	CTGGAGCGGATGATCGAGCG	OGR/ SGR4 semi-quantitative RT-PCR
BTBZ4_R	CTGGCGAGCTTCAGCATGTCC	OGR/ SGR4 semi-quantitative RT-PCR
BZIP50_F	GGTCGGCGCGGAGGATTTCT	OGR/ SGR4 semi-quantitative RT-PCR
BZIP50_R	ACTCCTGCATGGCTGTGGCG	OGR/ SGR4 semi-quantitative RT-PCR
ERF109_F	GGCTATCTTCAATTGTCACC	OGR/ SGR4 semi-quantitative RT-PCR
ERF109_R	GATTTATGTCGCCTGCTCTACC	OGR/ SGR4 semi-quantitative RT-PCR
HAK7 Intron2_R	TGCCACCAACTCCAGGCTGA	OGR/ SGR4 semi-quantitative RT-PCR
HAK7_Intron2_F	ATTACCGTGATGAGACAACC	OGR/ SGR4 semi-quantitative RT-PCR
HAK9 intron1_F	TTCCCAGCTCTCCTCGCCC	OGR/ SGR4 semi-quantitative RT-PCR
HAK9 intron1_R	ATGCCTCTCCCGAACTCAGG	OGR/ SGR4 semi-quantitative RT-PCR
SRDW5_R	ACCACCGGCCACCATGAGGT	OGR/ SGR4 semi-quantitative RT-PCR
SRWD5_F	TGCTCAATGTCGCGGGCCAA	OGR/ SGR4 semi-quantitative RT-PCR
TPP2_F	TGGAACAAGGGGAAAGCTGTGGA	OGR/ SGR4 semi-quantitative RT-PCR
TPP2_R	TCCTTGGGCACAGATGACACCA	OGR/ SGR4 semi-quantitative RT-PCR
WAK5_F	AGCGCGCACACGAATCAGCA	OGR/ SGR4 semi-quantitative RT-PCR
WAK5_R	ACAAGCATGGAGACGAAAATCACTGT	OGR/ SGR4 semi-quantitative RT-PCR

Name of the primer	Sequence (5' - 3')	Purpose
PHF_760_gRNA1_F	GGCACGTTTGTGCGAGAAATGTGA	PHF5A (LOC_Os04g56760) sgRNA cloning
PHF_760_gRNA1_R	AAACTCACATTTCTCGCACAAACG	PHF5A (LOC_Os04g56760) sgRNA cloning
PHF_760_gRNA2_F	GGCAAGACTCGGACAAGCGTACAT	PHF5A (LOC_Os04g56760) sgRNA cloning
PHF_760_gRNA2_R	AAACATGTACGCTTGTCCGAGTCT	PHF5A (LOC_Os04g56760) sgRNA cloning
PHF_410_gRNA1_F	GGCAAAGCGTACACGGGCGCACGT	PHF5A (LOC_Os05g30410) sgRNA cloning
PHF_410_gRNA1_R	AAACACGTGCGCCCGTGTACGCTT	PHF5A (LOC_Os05g30410) sgRNA cloning
PHF_410_gRNA2_F	GGCATGCGCCCGTGTACGCTTGTT	PHF5A (LOC_Os05g30410) sgRNA cloning
PHF_410_gRNA2_R	AAACAACAAGCGTACACGGGCGCA	PHF5A (LOC_Os05g30410) sgRNA cloning
PHF_760_F_1	GTTCGACCAACAGCTGAATAG	Genotyping of PHF5A (LOC_Os04g56760)
PHF_760_R_1	CGCCTTAAGTCCTTAACAACC	Genotyping of PHF5A (LOC_Os04g56760)
PHF_760_F_2	CATTGCTTCTCTTACACATACTATG	Genotyping of PHF5A (LOC_Os04g56760)
PHF_760_R_2	TCAGTGGCTTACATAATTATAAGAC	Genotyping of PHF5A (LOC_Os04g56760)
PHF_410_F_1	CACTTGAAAGTCTGTAGTCAG	Genotyping of PHF5A (LOC_Os05g30410)
PHF_410_R_1	CATACAAGTAACATTACAGCAAAC	Genotyping of PHF5A (LOC_Os05g30410)
PHF_410_F_2	CCTGACTGACTTATTACCAG	Genotyping of PHF5A (LOC_Os05g30410)
PHF_410_R_2	CTCATATTAGTATCAAAGCAGACC	Genotyping of PHF5A (LOC_Os05g30410)
3xFLAG-tag*PHF5A*-*Y36C*	ATGGACTATAAGGACCACGACGGAGACTACAAGGATCATGATATTGATTACAAAGACGATGACGATAAGGCAAAGCATCATCCTGATCTCATCATGTGCAGGAAGCAGCCTGGCATTGCTATTGGTCGTTTGTGCGAGAAATGTGATGGCAAGTGTGTCATCTGTGACTCGTGTGTGCGCCCATGTACGCTTGTCCGAGTCTGTGATGAGTGCAACTATGGTTCCTTCCAGGGGAGGTGTGTCATCTGTGGGGGGGTCGGCATCTCAGATGCCTACTACTGCAAGGAGTGCACTCAGCAGGAAAAGGACCGAGATGGATGTCCCAAGATTGTCAATCTTGGAAGCGCCAAGACCGATCTCTTCTATGAACGAAAGAAGTATGGTTTTAAGAAGAGATGA	Overexpression of mutated PHF5A (LOC_Os04g56760)_Y36C in rice

Supplementary Table 1: List of primers used for quantitative RT-PCR

MSUid	RAPDB_symbol	Fw primer	Rev primer
LOC_Os04g48850	ACS2	TCTCGCAGTTCAAGAGGATCGC	CATAAACTGGGCCATCGCCTTTC
LOC_Os01g72910	ASR4	ACAAGCACATGGAGCAGATCG	GCTTCTTCTCATGCATGGCGTATG
LOC_Os03g03550	BZIP25	TGAAGAAGATCATGGCCGACGAG	AGCTCCGCAATGTACCTCATCTTC
LOC_Os06g50830	BZIP56	AGAGAGAGAGAGAGAGCCAGAGAC	AGGATCCTTAATTCGATCGCCAAC
LOC_Os08g05960	DR10	AAGGAGAAGGCAGCTGCTAGTG	TAACCGGCACAGCAACGGAATC
LOC_Os02g19770	EIF1A1	TGTGATGTGTGAGGTCGTCTGC	ATCGCGTAAAGCGAGCAATTCG
LOC_Os11g06770	ERF118	TCGAGCAGATAATGGAGGACTCG	GAACAACGGAAACGACGACTGC
LOC_Os12g07030	ERF124	TCAACGGGTTCAGACGGAAGTG	GCATTTATTTCTGACGGCGTCTCG
LOC_Os09g28354	HSFB1	TGAGGTTGGTGGCAAGAATGGTC	TTGGCGTGCTTAATTCGTCTCG
LOC_Os01g60020	NAC4	CAAGAAGGGCTCACAGAAGCTG	ACCTTCTCCCAGTTGTTCTTCTTG
LOC_Os07g48730	RLCK241	CAGCAACTGCTCATCTGGGATTG	AGTTGCTGCTTCTCGTAACCTTTG
LOC_Os01g40430	WRKY27	AGAGAAGGTTCATCGGCATCACG	AGCCCAAGTGACAGGAATAACCC
LOC_Os06g44010	WRKY28	TCAAGAAGGATCACGAGGTTGAGG	ATCTCGGTCAGCTTCTTGTTCTCC
LOC_Os05g27730	WRKY53	TCCTCAGCTCCTCCCATATCTTG	TGCTGAGAAGCGATGAGATCGG
LOC_Os05g39720	WRKY70	TCCTCACGCCCAGTTTATTCCC	TCGTAATCCAGCTGAACAATGCG
LOC_Os06g06360	WRKY113	TGACACGATCAAGAGAAGGAAGGG	TGGGAATTTGGCACCGAGGATG
LOC_Os03g50885	Actin	TGGCATCTCTCAGCACATTCC	TGCACAATGGATGGGCCAGA
